# Association Between Fear and Beauty Evaluation of Snakes: Cross-Cultural Findings

**DOI:** 10.3389/fpsyg.2018.00333

**Published:** 2018-03-16

**Authors:** Eva Landová, Natavan Bakhshaliyeva, Markéta Janovcová, Šárka Peléšková, Mesma Suleymanova, Jakub Polák, Akif Guliev, Daniel Frynta

**Affiliations:** ^1^Department of Zoology, Faculty of Science, Charles University, Prague, Czechia; ^2^Applied Neurosciences and Brain Imaging, National Institute of Mental Health, Klecany, Czechia; ^3^Biology Faculty, Baku State University, Baku, Azerbaijan; ^4^Natural Historical Museum Named After Gasanbey Zardabi, Baku, Azerbaijan; ^5^Department of Psychology, Faculty of Arts, Charles University, Prague, Czechia

**Keywords:** attitude to snakes, cross-cultural study of emotions, envenoming, fear of snakes, perception of animal beauty, snakebites, viperidae

## Abstract

According to the fear module theory, humans are evolutionarily predisposed to perceive snakes as prioritized stimuli and exhibit a fast emotional and behavioral response toward them. In Europe, highly dangerous snake species are distributed almost exclusively in the Mediterranean and Caspian areas. While the risk of a snakebite is relatively low in Central Europe, Azerbaijan, on the other hand, has a high occurrence of the deadly venomous Levant viper (*Macrovipera lebetina*). We hypothesize that co-habitation with this dangerous snake has shaped the way in which humans evaluate snake species resembling it. For that purpose, we asked respondents from the Czech Republic and Azerbaijan to rank photographs depicting 36 snake species according to perceived fear and beauty. The results revealed a high cross-cultural agreement in both evaluations (fear *r*^2^ = 0.683, *p* < 0.0001; beauty: *r*^2^ = 0.816, *p* < 0.0001). Snakes species eliciting higher fear tend to be also perceived as more beautiful, yet people are able to clearly distinguish between these two dimensions. Deadly venomous snakes representing a serious risk are perceived as highly fearful. This is especially true for the vipers and allies (pit vipers) possessing a characteristic body shape with a distinct triangular head and thick body, which was found as the most fear evoking by respondents from both countries. Although the attitude toward snakes is more negative among the respondents from Azerbaijan, their fear evaluation is similar to the Czechs. For instance, despite co-habitation with the Levant viper, it was not rated by the Azerbaijanis as more fearful than other dangerous snakes. In conclusion, agreement in the evaluation of snake fear and beauty is cross-culturally high and relative fear attributed to selected snake species is not directly explainable by the current environmental and cultural differences. This may provide some support for the evolutionary hypothesis of preparedness to fear snakes.

## Introduction

Detection and an appropriate reaction to dangerous animals and other life threatening stimuli were necessary for human survival (Barkow, 1992; New et al., 2007). Even in contemporary humans, animal species receive considerably more attention over other stimuli (Altman et al., 2016; Calvillo and Hawkins, 2016; but see Hagen and Laeng, 2016). For example, people are able to rapidly detect various potentially dangerous animals eliciting fear (Tipples et al., 2002; Yorzinski et al., 2014). Öhman and Mineka (2001) proposed the existence of an evolutionary fear module, a complex system consisting of neural, psychophysiological, and behavioral reactions to potentially life-threatening stimuli, particularly those evolutionary relevant. However, all stimuli evoking emotions, in general, attract attention more than neutral stimuli (Vuilleumier, 2005).

Of all the dangerous animals that human ancestors frequently encountered and feared in their environment, snakes have been used as an example of a prototypical stimulus activating the hardwired neural circuitry of fear (Öhman and Mineka, 2003) and their role as a significant selection pressure seems plausible. Throughout the course of evolutionary history venomous snakes presented a real threat (Öhman and Mineka, 2001; Öhman et al., 2001; Isbell, 2006; LoBue and DeLoache, 2010; LoBue and Rakison, 2013) requiring a rapid detection. This drove human ancestors to evolve attentional bias to snakes and similar survival endangering stimuli (Lipp and Waters, 2007). This has been demonstrated in visual search tasks when both adults, as well as 3-year-old children, visually detected snakes more rapidly than other kinds of stimuli (LoBue and DeLoache, 2011). Interestingly, reactions to snake stimuli are even faster than to spiders (Soares et al., 2009), which are both common phobic objects (Davey, 1994). It is the snake body shape (LoBue and DeLoache, 2011) and bright colors (Hayakawa et al., 2011; Landová et al., 2012) together with other typical snake characteristics (LoBue, 2014; Janovcová, 2015) that enhance the fast and accurate visual detection (LoBue et al., 2014). High attentional bias combined with innate (Weiss et al., 2015) or learned fear of snakes (Mineka et al., 1980; Cook et al., 1985; Cook and Mineka, 1989) in many primates including humans allow for quick associations between snake cues in the environment and an appropriate behavioral reaction to the imminent threat (Van Le et al., 2013; LoBue, 2014).

Tierney and Connolly (2013) note in their comprehensive review that, due to methodological reasons, ontogenetic and comparative studies of snake fear are not conclusive and fully comparable. Thus, we are not certain if snake fear has an evolutionary basis only. However, Van Le et al. (2013) reported the existence of neurons in the medial and dorsolateral pulvinar of macaques that responded faster and stronger to snake stimuli than to monkey faces and other objects. These results further support the evidence that primates (Van Le et al., 2013, 2014) as well as humans (Van Strien et al., 2014; Almeida et al., 2015) possess a neurobiological substrate for a rapid detection of snakes as threatening visual stimuli. They also corroborate the evolutionary perspective that snakes have shaped the evolution of the visual system in the primate lineage (Isbell, 2006). The question remains, however, to what extent these evolutionary based attentional biases coincide with the current level of danger represented by snakes in specific geographical regions and how these both factors influence emotions evoked by snakes.

Some colubroid snakes in Africa and Asia have evolved a potent venom delivery system (Vidal and Hedges, 2002); the highly venomous snakes include viperids and elapids (Fry et al., 2006). Interestingly, this innovation coincided with the appearance of rodents and primates (reviewed in Isbell, 2006). For example, it is now agreed that viperids and anthropoid primates evolved together in the Old World and thus share a long history of co-evolution (Keogh, 1998; Beard, 2002; Miller et al., 2005). The major threat pertaining to snakes is currently the risk of envenoming especially in some regions like South and Southeast Asia and Sub-Saharan Africa (Swaroop and Grab, 1954; Chippaux, 1998). Nowadays, venomous snakebites are still an important cause of human injuries and even deaths in some countries. A global estimate for the occurrence of envenomings per year is 421,000–1,841,000 cases resulting in 20,000–90,000 deaths (Kasturiratne et al., 2008). However, the statistical estimates are based on records reported by doctors or data about compensation for the farming community (various accidents including snakebites). Even if all these factors are taken into account, the data might still be underestimated for developing countries or for regions where no exact information about snakebites epidemiology and subsequent injuries is available. Besides that, constrictor non-venomous snakes were for a long time significant predators of primates and human ancestors and even now may represent a serious danger to humans. It has been shown on Agta Negritos, a preliterate society of hunter-gatherers in the Philippines, that 26% of adult males survived predation attempts by large constrictors, specifically the reticulated python (Headland and Greene, 2011).

**Figure 1:**
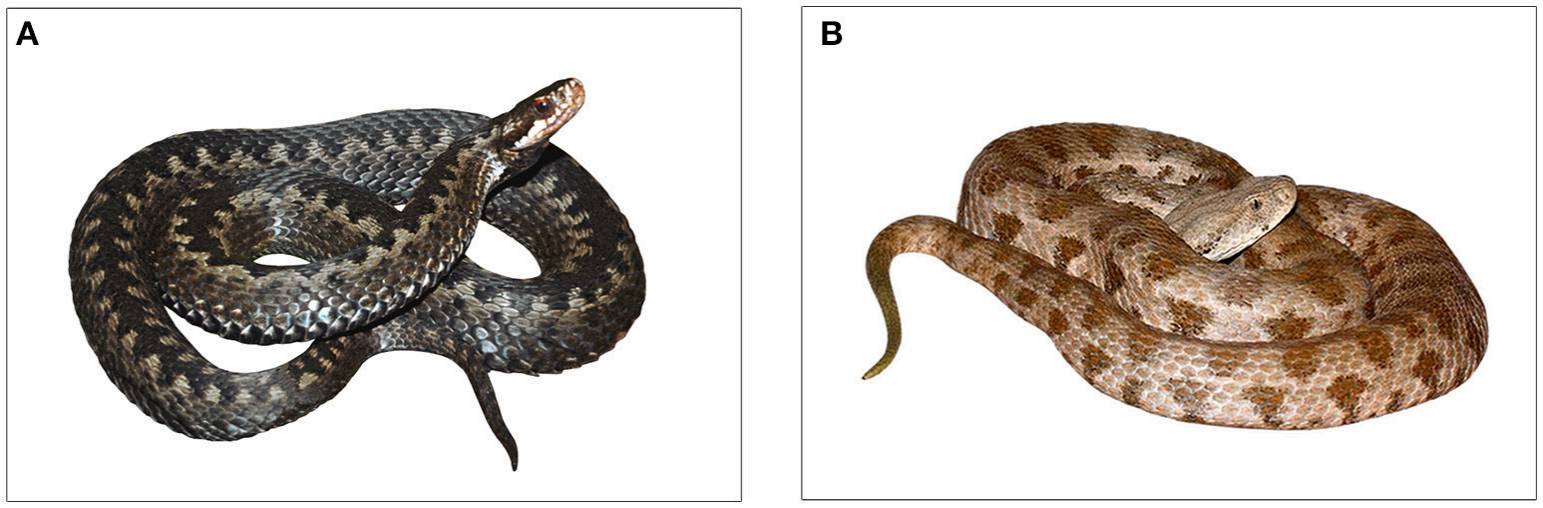
The most dangerous species of venomous snakes in each country: **(A)** the common European adder (*V. berus*) from the Czech Republic, original photo Pavel Kirillov, standardized version; **(B)** the Levant viper (*M. lebetina*) from Azerbaijan, original photo Omid Mozaffari, standardized version. We predict that these snakes should elicit the highest fear in the Czech Republic and Azerbaijan, respectively.

The danger of a particular snake species for humans varies with the efficiency of venom and its delivery system, the snake's size, aggressiveness, and the probability of encounter with humans. In North, Central, and Western Europe, only small venomous species of vipers can be found, such as the common European adder (*Vipera berus*, Figure 1A). More dangerous is the nose-horned viper (*Vipera ammodytes*), but its distribution is mainly in South-Eastern Europe. Envenoming by the common adder results in tachycardia, dizziness, hypotension, shock, and gastrointestinal symptoms, coagulopathy and neutrophil leucocytosis (Malina et al., 2008). However, in the last 20 years, there has be no reported death caused by this snake in the Czech Republic (Valenta, 2008). Contrastingly, highly dangerous snake species of Europe are distributed almost exclusively at its eastern border, in the Caspian region (Chippaux, 2012). The most important snake species causing the most of envenomings and subsequent deaths in Azerbaijan is the Levant viper (*Macrovipera lebetina*, Figure 1B). Bites of the Levant viper are far more dangerous compared to the common adder and provoke serious symptoms such as oedema, hypotension shock, hemorrhage, tissue necrosis, and melanoderma (Göçmen et al., 2006). In Azerbaijan, 106 cases of bites and one death caused by this species were reported since April to October in 2016 (Bakhshaliyeva, unpublished data); for more information about snakes in this region (based on Bannikov et al., 1977; Coborn, 1991; Schultz, 1996; Amr et al., 1997; Khan, 2002; Spawls et al., 2002; Marais, 2004; El Din, 2006; Egan, 2007; Valakos et al., 2008; Stojanov et al., 2011; Wallach et al., 2014) and their dangerousness (based on Brown, 1973; Weiser et al., 1984; Spawls et al., 1995; Mallow et al., 2003; Abdel-Aal and Abdel-Baset, 2010; Weinstein et al., 2011; Nalbantsoy et al., 2012, 2013; Hossie et al., 2013; Yousefkhani et al., 2014; Samy et al., 2015; Steinhoff, 2017) see Supplementary Tables 1,2.

Snakes are an ambivalent stimulus perceived as a source of actual or evolutionary fixed danger that attracts specialized attention and triggers fear. Together with a negative picture caused by the media, this may result in negative attitudes toward snakes in both adults (Pandey et al., 2016) and children (Yorek, 2009; Ballouard et al., 2013), including snake killing (Pandey et al., 2016). It has been demonstrated that more negative attitudes toward snakes may be associated with a higher risk of snakebite in the specific region. In South-Eastern Australia, for example, where most of the snakes are highly venomous, one-third of encountered snakes are killed by local people (Whitaker and Shine, 2000). However, especially children attitudes toward snakes vary in different countries and may be modified by targeted educational programs (Ballouard et al., 2012, 2013). On the other hand, snakes are seen as positive divinities in some countries, a source of meat, or a part of traditional medicine (Alves et al., 2012).

In contrast to the cross-cultural variation in negative attitude toward snakes (Ballouard et al., 2013), evidence shows that humans express a universal, cross-cultural agreement in how they evaluate beauty of some snake species (Frynta et al., 2011) which might be closely connected with positive emotions such as joy (see a meta-analysis by Kühn and Gallinat, 2012). Although perceived beauty and fear of snakes may correlate (Janovcová, 2015), these two form distinct dimensions so beauty of a snake cannot fully explain the level of fear it evokes, as we found in king snakes (Landová et al., 2012).

Here we hypothesize, that despite the evolutionary preparedness model, the risk of a snakebite in a given locality is an important predictive factor modulating negative or positive evaluation of snakes. People living in a region with a low abundance of dangerous snakes might have less negative attitudes toward them and experience fear of snakes differently than people living in a country where venomous snakes are more numerous. Therefore, the relationship between the perceived danger, emotions underlying the evaluation of fear, and attitudes toward snake species in different countries warrant future research. To study this effect, we recruited participants from two countries with a considerably different risk of envenoming. Only five species of snakes live in the Czech Republic, four of them are non-venomous and only one presents a potential though moderate danger to humans. Contrary to that, 32 species of snakes live in Azerbaijan and some of those are deadly venomous.

The aims of this study are as follows:

1. As the risk of a bite by venomous snakes is considerably lower in the Czech Republic compared to that in Azerbaijan (the area with a high occurrence of the deadly venomous Levant viper), we expect that the fear response will also be qualitatively different in these two countries. We hypothesize that co-habitation with the highly dangerous viper in Azerbaijan shaped human fear evaluation of snake stimuli resembling it.
2. Furthermore, some animals may trigger more than one emotion at a time, e.g., fear linked to a potential threat and pleasure associated with the perception of animal beauty (Landová et al., 2012). Both fear and perceived beauty could influence the way we view animals, and therefore, we have decided to include the evaluation of snake beauty as a control task to fear rankings. This would allow us to disentangle these two judgements and identify whether snake fear and beauty evaluations form two separate dimensions or are in any way interlinked through a general categorization process as we have previously found in the king snakes (Landová et al., 2012). Contrary to fear, we expect a high cross-cultural agreement in snake beauty evaluation, similarly to already reported results on boid snakes (Marešová et al., 2009a; Frynta et al., 2011).
3. An important question that remains unstudied is what accounts for human evaluation of specific snake species as fear or beautiful evoking. Here we suggest that specific features of snakes' appearance lead to their evaluation as “dangerous” and more fear evoking while other features are linked to the positive evaluation of snake beauty. In the study reported here, we examined morphological (body form), color, and other visual features of snake photo stimuli that account for specific fear and beauty ranking.
4. Evaluation of animals by humans might be influenced by sociodemographic factors such as formal education and gender or previous personal experience. In this study, we could analyze the effect of gender and type of education (biological vs. non-biological) on human fear and beauty evaluation of snakes.
5. Finally, as the risk of a venomous bite influences attitudes toward snakes in some countries (Ballouard et al., 2013; Pandey et al., 2016), we also asked our respondents in the Czech Republic and Azerbaijan how much they liked snakes and if they have experienced snake killing. If the attitude was similarly negative in both countries, then we could assume that humans possess an innate “fear module” influencing their mental representation of snakes as dangerous animals, even though some of them (especially the Czech respondents) have never risked a venomous snakebite in their life.

## Materials and methods

### Participants

In total, 104 individuals (50 men and 54 women) from the Czech Republic (CZ) and 100 individuals (38 men and 62 women) from Azerbaijan (AZ) were included in the study. We recruited the Czech respondents among undergraduate students from Charles University and University of South Bohemia and their families. Similarly, the Azerbaijani participants were recruited among the undergraduate students and staff of Baku State University together with their families. This has been driven by the need for a homogenous and comparable sample of respondents in both countries that would be attentive enough to complete the task (see the Discussion for more details).

The mean age of Czech respondents was 30.00 ± 1.67 and 30.46 ± 1.45 years, for men and women, respectively. Similarly, the mean age of men and women respondents from Azerbaijan was 31.16 ± 2.46 and 20.79 ± 1.67, respectively. More than a half of the respondents completed a bachelor degree in biology (55 Czechs and 78 Azerbaijanis) or another university degree, only 12 Azerbaijani and 28 Czech individuals had a secondary high school education. All subjects gave their written informed consent in accordance with the Declaration of Helsinki and then filled a questionnaire written in their native language (Czech or Azeri) asking for their gender, age, family membership, attitude toward snakes, and experience with them (see Supplementary Table 3 for an English translation of the form and more details below). The study has been approved by the research ethics committee of Charles University and National Institute of Mental Health.

### Attitude toward snakes

Based on the most frequent spontaneous responses given in our previous research in the Czech Republic and Azerbaijan, attitude toward snakes was measured using a 7-point Likert scale from 1 (“I like snakes, I would like to breed them at home”) through 4 (neutral) up to 7 (“I hate snakes/I fear them). We also asked the respondents whether they have ever encountered a real snake and if so, to give more details (“How and where have you met snakes?”). We also asked if they have ever killed a snake or seen someone else killing a snake. The responses were coded as either 1 = Yes or 0 = No.

### Stimuli

We examined a list of extant snake species inhabiting Europe, the Mediterranean and Caspian area (Uetz et al., 2015). For this study, we selected 36 species covering the main morphological diversity and all major phylogenetic lineages. The included species belong to 6 families and 29 genera (Pyron et al., 2013; Uetz et al., 2015; for more information about the tested snakes, see Supplementary Table 1). To test perceived fear and beauty, we developed a standardized set of 36 photographs depicting these species, each representing a typical adult individual in a resting position. In addition, we included a control photograph of the Egyptian cobra (*Naja haje*) in a striking posture for a comparison with the resting position picture. The photos were adapted from books and internet sources (for sources and other information, see Supplementary Table 4). We digitally set the pictures of snake bodies on a white background, resized them to a comparable size (regardless of their real size) using GIMP2.8.16 (GNU Image Manipulation Program. Spencer Kimball, Peter Mattis et al., 1995 – 2015), and printed them in a 10 × 15 cm format. It has been previously demonstrated that photos may reliably substitute live snakes (Landová et al., 2012).

### Stimuli ranking according to fear and beauty

The respondents were instructed to sort the photographs of live snakes so that the “most beautiful” species would be on the top of the pack, the second “most beautiful” under, etc., until the last selected species at the bottom of the pack. After a short break (~5 min) they were asked to sort the photographs again, this time according to perceived fear. No time limit was set for performing both tasks. Each photograph's rank in the pack according to fear and beauty provided by the respondents was further examined. The ranks were square-root arcsin-transformed to increase the importance of the distribution tails and to improve normality. Mean values of the transformed ranks were used further as a scale for perceived fear and beauty of snakes (the higher the value the lower the perceived fear or beauty). These fear and beauty scales were calculated separately for the Azerbaijani and Czech respondents and further correlated to assess similarities in the fear and beauty evaluations of snakes in the two countries.

### Extraction of morphological and color characteristics

We measured morphological characteristics of the depicted species, specifically the body length, head width and length, tail width, neck width, and the eye diameter, using the software Image Tool 3.1 (Wilcox et al., 2002). All the values were measured in millimeters and log-transformed for further analyses. Color properties of the stimuli, namely the proportional ratio of chromatic and achromatic colors, the mean and standard deviation of lightness and saturation and the complexity of lightness pattern, were extracted using the software Barvocuc (for a more detailed description of the software see Lišková and Frynta, 2013; Lišková et al., 2015; Rádlová et al., 2016). The hue values (defined in the HSL color space) for chromatic colors were pre-defined using the following angles: red (<330°; 17°), orange/brown (<17°; 39°), yellow (<39°; 67°), green (<67°; 180°), blue (<180°; 260°), purple (260°; 310°), and pink (<310°; 330°). Values for achromatic colors (with values of 0-1 in the HSL color space) corresponded to the following setting: white (L > 0.80), black (L < 0.28), and gray (S < 0.10). The pattern complexity was computed in Barvocuc using the Sobel operator (Sobel, 1978); minimum 0.2013, e.g., a smooth snake as the European blind snake (*Xerotyphlops vermicularis*), maximum 0.8452, e.g., a snake with a complex pattern with pronounced scales, such as the common European adder or the African puff viper (*Bitis arietans*). For the graphical output from the Barvocuc program, see Supplementary Figure 1. To improve normality, we square-root arcsin-transformed the values of color representations and pattern complexity (both expressed as a proportion of the area covered by the animal in the picture) for further analyses. Furthermore, we extracted the area covered by the animal in the picture (the number of non-transparent pixels transformed to square millimeters and square rooted).

### Statistical analyses

Agreement among the respondents was quantified by the Kendall's coefficient of concordance (W) as implemented in SPSS (Statistical Package for the Social Sciences), version 16.0 (Chicago, IL, USA). Next, the transformed data (square-root arcsin-transformed) were analyzed by the Principal Component Analysis (PCA) to visualize their multivariate structure. We computed two-way, consistency average score Intra-class Correlations (ICC, Hallgren, 2012) to estimate reliabilities of mean transformed ranks (command icc, irr package, in R; R Development Core Team, 2010). The cross-cultural agreement in fear and beauty and a correlation between both evaluations were analyzed by the least square linear regression (Pearson r-squared). To test which respondent's characteristic (sex, age, nationality, attitudes toward snakes and their interaction) influence the evaluation of beauty and fear, we used MANOVA (Multivariate Analysis of Variance); a final reduced model was built from an initial full model by backwards removal of the factors which appeared non-significant.

A different model was used for testing the effect of family membership and respondents' individual characteristics on the attitude itself. We computed a marginal model (command gls as implemented in package nlme in R software), testing the effect of nationality, sex, age, type of education and nationality*sex interaction. We computed one model with and another one without the random effect of family membership. Subsequently, these two versions of the model were compared using ANOVA. We also performed Canonical Discrimination Function Analysis (DFA, forward stepwise model) for the fear and beauty evaluations to identify those species whose evaluations matched/differed significantly. Next, we performed GLMs (Generalized Linear Models) to analyze the effect of traits of the assessed snakes, i.e., morphological and color characteristics (see above) on their beauty and fear evaluation. To calculate correlations between the fear eliciting evaluation and a specific level of threat to humans we used the Spearman's rank correlation coefficient (Spearman R). All those analyses, if not stated otherwise, were performed in Statistica 8.0 (StatSoft Inc., 2010).

## Results

### Congruence among respondents

First, we evaluated agreement among the respondents for each country and evaluation separately. Kendall's coefficient of concordance (W) showed sufficient agreement among the respondents from the Czech Republic for fear (*N* = 104, W = 0.341, *p* < 0.0001) and beauty evaluation (*N* = 104, W = 0.201, *p* < 0.0001). The concordance coefficients computed for Azerbaijan respondents were also significant (fear: *N* = 100, W = 0.180, *p* < 0.0001; beauty: *N* = 100, W = 0.263, *p* < 0.0001). When the picture of the Egyptian cobra (*N. haje*) in the threatening posture was excluded from the datasets, the agreement in fear evaluation remained significant in both Czech (*N* = 104, W = 0.315, *p* < 0.0001) and Azerbaijani respondents (*N* = 100, W = 0.151, *p* < 0.0001).

In order to estimate reliabilities of mean transformed ranks, we computed average score intra-class correlations (ICC). The resulting population values were high in both Czech [N_stimuli_ = 37, N_raters_ = 104, fear: ICC = 0.983, 95% CI = 0.974–0.990, *F*_(36, 3708)_ = 59.4, *p* < 0.0001; beauty: ICC = 0.967, 95% CI = 0.950–0.980, *F*_(36, 3708)_ = 30.3, *p* < 0.0001] and Azerbaijan datasets [N_stimuli_ = 37, N_raters_ = 100, fear: ICC = 0.958, 95% CI = 0.936–0.975, *F*_(36, 3564)_ = 23.8, *p* < 0.0001; beauty: ICC = 0.975, 95% CI = 0.962–0.985, *F*_(36, 3564)_ = 40.2, *p* < 0.0001].

### Respondents' characteristics affecting fear and beauty evaluation of snakes

To test for the effect of respondents' individual characteristics (biology education, age, sex, nationality, and interactions thereof) on the fear and beauty evaluations, we employed MANOVA. For fear, there was no significant effect of biology [*F*_(164, 35)_ = 1.36, *p* = 0.1025, power = 0.97], age [*F*_(164, 35)_ = 0.63, *p* = 0.9486, power = 0.61], nor sex [*F*_(164, 35)_ = 0.99, *p* = 0.4907, power = 0.87], however there was a significant effect of nationality [*F*_(164, 35)_ = 4.65, *p* < 0.0001, power = 1.00] and nationality*sex interaction [*F*_(164, 35)_ = 2.03, *p* = 0.0017, power = 1.00]. For beauty, we found a significant effect of nationality [*F*_(164, 35)_ = 4.13, *p* < 0.0001, power = 1.00], and age [*F*_(164, 35)_ = 1.68, *p* = 0.0162, power = 0.99], while biology [*F*_(164, 35)_ = 1.48, *p* = 0.0551, power = 0.98], sex [*F*_(164, 35)_ = 1.13, *p* = 0.2989, power = 0.92], and nationality*sex interaction [*F*_(164, 35)_ = 1.49, *p* = 0.0528, power = 0.98] were not significant. We further tested the effect of family membership as a random factor on fear and beauty scores in the two most prominent species in both countries, i.e., the common European adder (Vipera) and the Levant viper (Macrovipera). Two versions of a marginal model (command gls as implemented in package nlme in R software) with and without the family factor were compared by ANOVA. It appeared that neither fear nor beauty evaluation was effected by the family membership as having included it into the model did not result in a significant improvement (Macrovipera-fear: likelihood ratio = 1.67, *p* = 0.1961; Vipera-fear: likelihood ratio = 1.21, *p* = 0.2720; Macrovipera-beauty: likelihood ratio = 0.11, *p* = 0.7401; Vipera-beauty: likelihood ratio = 0.65, *p* = 0.4213).

### Attitude toward snakes and experience with killing a snake in czech and azerbaijani respondents

We tested the effect of individual characteristics (nationality, sex, type of education, and nationality*sex interaction) and family membership on the attitude toward snakes. Using the same type of analysis as mentioned above, i.e., creating two marginal models with and without the family membership as a random factor and comparing them by ANOVA, it was revealed that the model with the family membership was significantly better (likelihood ratio = 8.72, *p* = 0.0033; AIC of the model with and without the family membership: 816.04 vs. 823.05, respectively). The resulting model further showed a significant effect of nationality [*F*_(1, 199)_ = 27.44, *p* < 0.0001] and sex [*F*_(1, 199)_ = 17.34, *p* < 0.0001], but not the nationality*sex interaction [*F*_(1, 199)_ = 0.75, *p* = 0.3879] nor biology education [*F*_(1, 199)_ = 3.29, *p* = 0.0714].

Different attitude toward snakes in both countries could be further demonstrated by responses on the provided measure. In the Czech Republic, 54% of respondents had a positive attitude toward snakes, 20% neutral, and 26% a negative one. In Azerbaijan, only 14% of respondents had a positive attitude toward snakes, 49% neutral, and 37% a negative one. Interestingly, the incidence of snake encounters was comparable in both countries (99% of Czechs and 89% of Azerbaijanis have met a snake, *p* = 0.0023), even though the number of snake species living in the respective country is strikingly different (see the Introduction).

We also tested the effect of individual characteristics on experience with killing a snake or having seen someone killing one. We computed a GLM model (for binomial data with logit link function) with nationality, sex, nationality*sex interaction, and biological education as fixed factors. The frequency of killing a snake was influenced only by nationality (*Z* = −3.60, *p* = 0.0003); more respondents in Azerbaijan than in the Czech Republic have killed a snake or have seen someone killing it (49% vs. 14%). Moreover, people in the Czech Republic reported having this experience mostly as a part of veterinary care or road accident.

When using MANOVA to analyze the additive effect of reported attitude toward snakes and experience with snake killing in both countries on fear evaluation, both the nationality (*F* = 3.16, *p* = 0.0001, power = 0.99) and snake killing influenced it significantly (*F* = 1.62, *p* = 0.0252, power = 0.99), but no significance was found for the attitude toward snakes (*F* = 1.00, *p* = 0.4834, power = 1.00). A similar statistical model for beauty evaluation was influenced only by nationality (*F* = 2.54, *p* = 0.0001, power = 0.99), but neither the attitude (*F* = 1.15, *p* = 0.0895, power = 1.00) nor snake killing (*F* = 1.48, *p* = 0.0562, power = 0.98) were significant.

### Are fear and beauty two separate dimensions?

In the Czech respondents, perceived fear positively correlated with beauty (*r*^2^ = 0.379, *p* = 0.0001), however this correlation was insignificant in Azerbaijanis (*r*^2^ = 0.063, *p* = 0.1415). Such difference could be explained by the MANOVA results (see section Respondents' Characteristics Affecting Fear and Beauty Evaluation of Snakes above) showing that there was a significant difference in the rankings between men and women, but only in the Azerbaijani sample. Therefore, we analyzed the relationship of perceived fear and beauty for each sex and country separately. In Czech men, the correlation was highly significant and stronger (*r*^2^ = 0.424, *p* < 0.0001) than in Czech women (*r*^2^ = 0.299, *p* = 0.0006). In Azerbaijani men, the correlation was rather weak, yet significant (*r*^2^ = 0.170, *p* = 0.0125). However, in Azerbaijani women, there was no significant correlation (*r*^2^ = 0.012, *p* = 0.5290). These results suggest that the correlation between fear and beauty in Azerbaijani respondents is influenced by the different precision in fear rankings (the highest fear/beauty discrepancy was found in the Azerbaijani women, followed by the Azerbaijani men, Czech women, and Czech men) and the difference in experience with snake killing (see above). Nonetheless, when the data from Azerbaijan were analyzed separately, the sexes correlated significantly in both fear (*r*^2^ = 0.746, *p* < 0.0001) and beauty evaluation (*r*^2^ = 0.866, *p* < 0.0001).

### Correlation between czechs and azerbaijanis in fear and beauty evaluation

**Figure 2:**
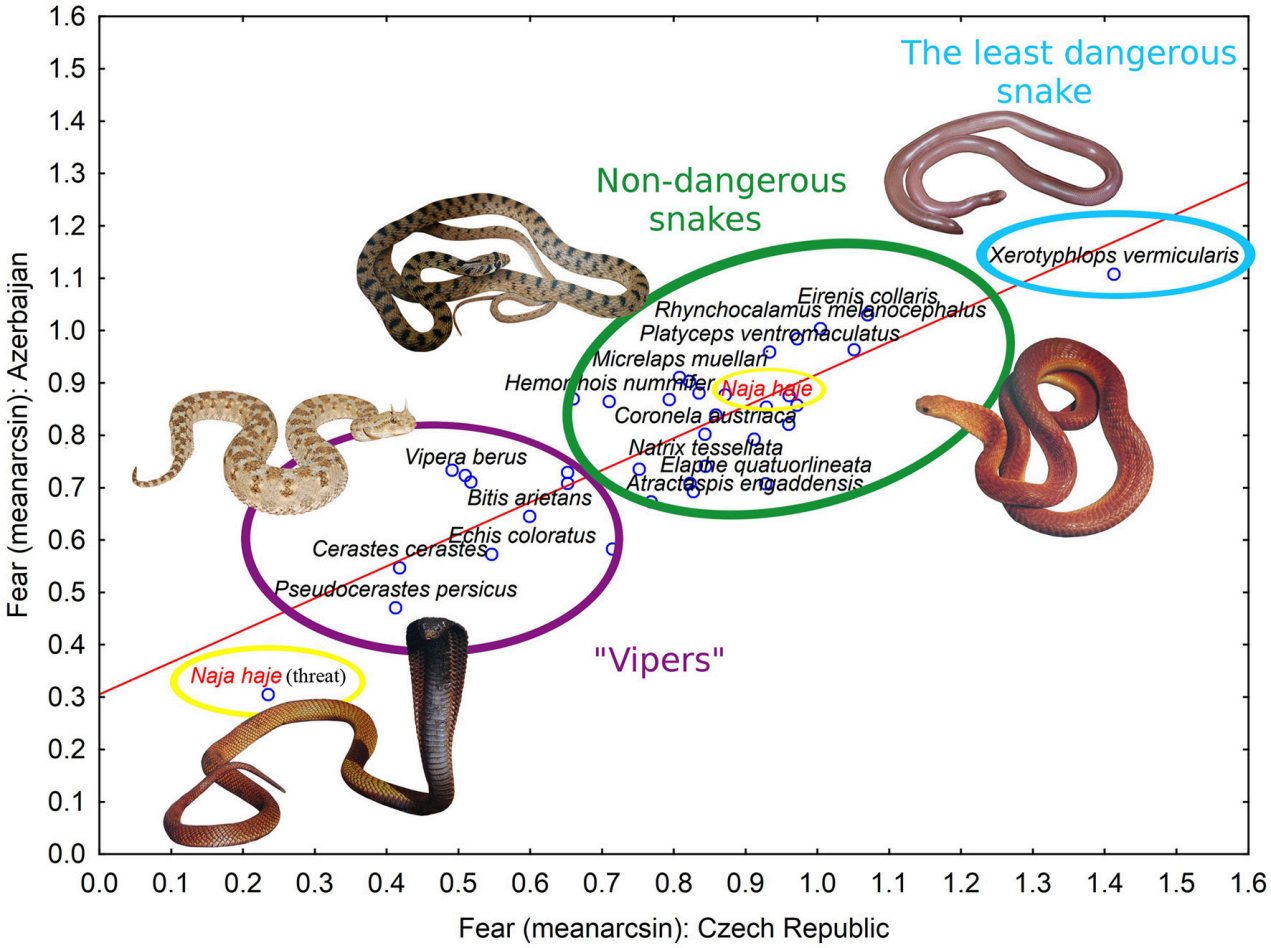
A correlation of fear evaluations from the Czech Republic and Azerbaijan. The graph was calculated from square-root arcsin-transformed data, i.e., the lower the value the more fearful the species is according to the respondents (Pearson correlation coefficient; *r*^2^ = 0.683, *p* < 0.0001). The highlighted evaluation of the Egyptian cobra in two positions illustrates different perception of snakes depending on their posture.

In section Respondents' Characteristics Affecting Fear and Beauty Evaluation of Snakes, we detected a significant effect of nationality on mean ranks of fear and beauty. Despite that, a comparison of photos ranking in both countries according to a particular evaluation showed a high cross-cultural agreement (Pearson correlation coefficient; fear: *r*^2^ = 0.648, *p* < 0.0001, Figure 2; beauty: *r*^2^ = 0.832, *p* < 0.0001, Supplementary Figure 2). The remaining variability in the data may be explained by the statistical models we have used to analyze the effect of demographic variables and attitude toward snakes. For the complete results of fear and beauty evaluations for particular species, see Supplementary Table 5.

**Figure 3:**
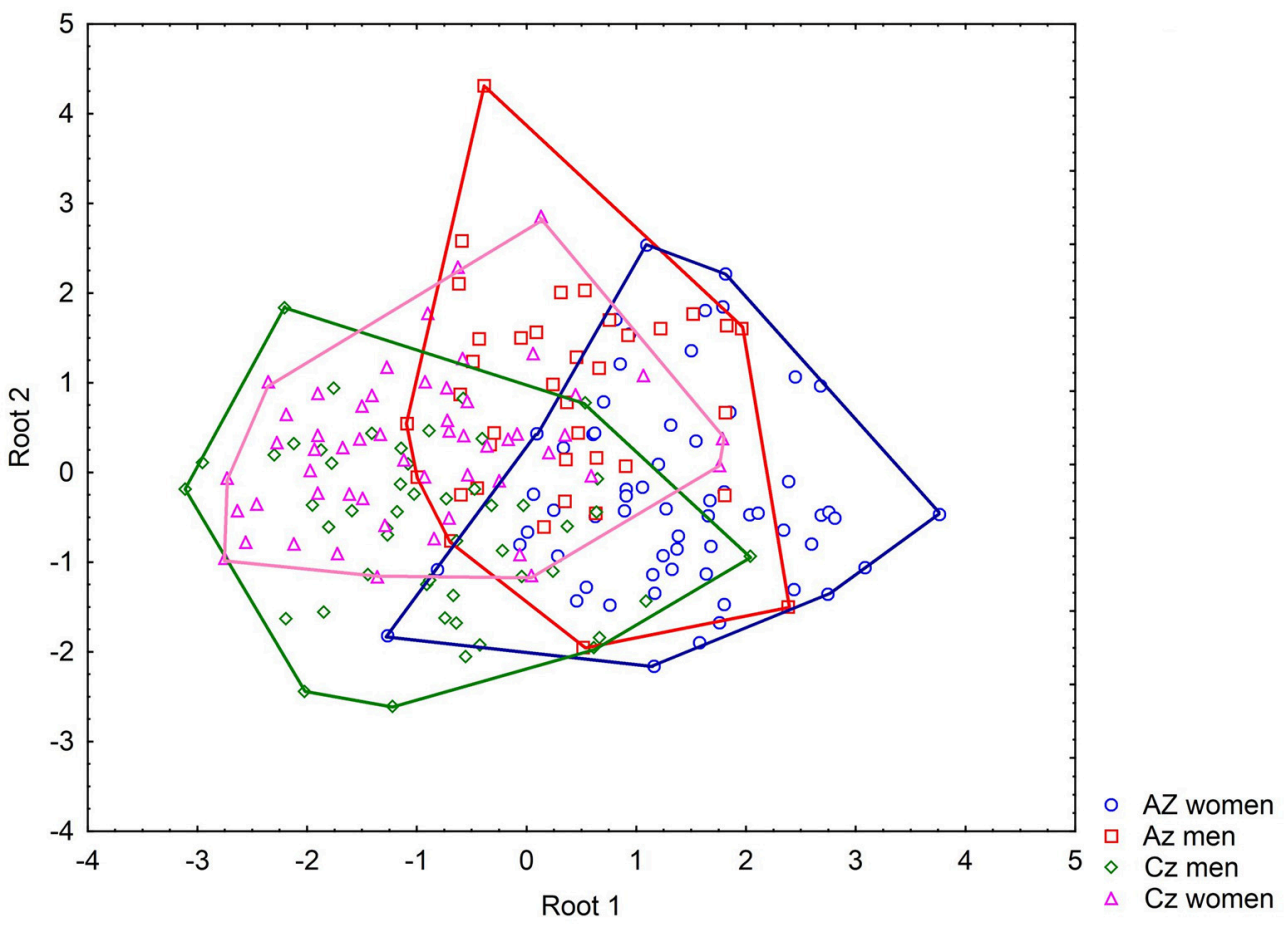
The canonical DFA of fear evaluation. This analysis showed significant differences between the sexes and countries when comparing the Azerbaijani women to all other groups and the Azerbaijani men to the Czech respondents. However, a plot of the first two canonical factors showed a considerable overlap of the groups for elicited fear. The number of included species was reduced by the forward stepwise procedure to just 17 out of 36. Three species, the meadow viper (*Vipera ursinii*; Wilks' lambda = 0.3850, *p* = 0.0042), the coastal viper (*Montivipera xanthina*; Wilks' lambda = 0.3975, *p* = 0.0002), and the Palestine saw-scaled viper (*Echis coloratus*; Wilks' lambda = 0.3898, *p* = 0.0014) had the largest Wilks' lambda and therefore, corresponded the best with the discrimination criteria. Thus, only three of the 36 tested snake species are responsible for the detected differences in fear evaluation.

We also performed a canonical discrimination function analysis (DFA) to assess differences/overlaps between the groups of respondents (nationality and gender) in the fear evaluation [Wilks' lambda = 0.36; *F*_(51, 548)_ = 4.43; *p* < 0.0001] of selected 17 species, see Figure 3 for details. Even though the analysis was significant, the classification success was rather low (62.25%), which signifies an overlap between the groups. This analysis showed significant differences between the sexes and countries when comparing the Azerbaijani women to the remaining groups (Azerbaijani men: *F* = 8.95, *p* = 0.0031; Czech men: *F* = 33.14, *p* < 0.0001; Czech women: *F* = 39.37, *p* < 0.0001) and the Azerbaijani men to the Czech respondents (Czech men: *F* = 4.87, *p* = 0.0284; Czech women: *F* = 6.70, *p* = 0.0103). However, there was no significant difference when comparing the Czech men and women (F = 0.14, *p* = 0.71). A plot of the first two canonical factors showed a considerable overlap of the groups (see Figure 3, loadings are provided in Supplementary Table 6).

Similarly, an analogical analysis was performed to assess differences/overlaps between the groups of respondents (ethnic and gender) in their evaluation of snake beauty [Wilks' lambda: 0.3390, *F*_(108,495)_ = 2.00, *p* < 0.0001, 24 species were included, see Supplementary Figure‘3]. The classification success was again rather low (62.75%) signifying an overlap between the groups. As with the analysis of fear evaluation (see above), there was no difference between the Czech men and women (*F* = 1.17, *p* = 0.2749). Contrary to that, the Azerbaijani women were different from all the other groups again (Azerbaijani men: *F* = 2.58, *p* = 0.0002; Czech men: *F* = 3.69, *p* < 0.0001; Czech women: *F* = 3.51, *p* < 0.0001) and the Azerbaijani men differed from the Czech participants (Czech men: *F* = 3.70, *p* < 0.0001; Czech women: *F* = 2.58, *p* = 0.002). Still, a plot of the first two canonical factors showed a considerable overlap of the groups (see Supplementary Figure 3, loadings are provided in Supplementary Table 7).

### Explanation of perceived fear and beauty by physical traits of snake stimuli

Because of the high correlation of snake rankings according to fear and beauty between the Czech and Azerbaijan respondents (see above), we computed a model explaining the influence of snake morphology and color from a combined data set (CZ and AZ respondents pooled together), but separately for beauty and fear rankings. We computed optimal linear models (LM), which we used to analyze the effect of morphological and other perception characteristics (color, pattern, lightness, saturation) of stimuli on the fear and beauty evaluations.

The optimal model (LM) for the perceived fear explained 83.6% of variability. There was a positive effect of the head width, eye width, tail width, and white color on high fear evaluation. We found a negative effect of the overall body length, photograph lightness and pink color that reduce fear evaluation of snakes (see Table 1). The optimal model (LM) for the perceived beauty explained 80.7% of variability. We found a positive effect of the pattern complexity, amount of black color, and overall lightness of the photograph that all increase the snake beauty evaluation. On the contrary, white and red colors of snakes had a negative effect and decreased the beauty evaluation. Interestingly, none of the morphological characteristics had a significant effect on beauty (see Table 2). Dark, short, thick snakes with a wide head and big eyes elicit the highest fear, while snakes with complex disrupted patterns and bright colors are evaluated as the most beautiful. Thus, different sets of morphological and color characteristics influence the fear and beauty evaluation, but only lightness of the snake picture stimuli influences the fear (dark snakes are the most fear eliciting) as well as beauty (brightly colored snakes are the most beautiful) evaluation.

**Table 1:** Final reduced model (LM) describing the effects of snake morphology and color on fear ranking.

	**Df**	***F*-value**	**Pr(>*F*)**	**Estimate**	**SE**	***t* value**	**Pr(>|*t*|)**
Intercept				0.171	0.492	0.347	0.7312
Body length	1			0.148	0.070	2.098	0.0458
Tail width	1					−2.546	0.0172
Head width	1	26.99	< 0.0001	29.180	< 0.001	−2.947	0.0067
Eye width	1	10.21	0.0036	11.870	0.002	−2.585	0.0157
Mean lightness	1	18.86	0.0002	1.435	0.296	4.851	< 0.0001
SD lightness	1	0.24	0.6267	1.738	0.529	3.287	0.0029
White	1	29.52	< 0.0001	−1.018	0.220	−4.628	< 0.0001
Purple	1	0.01	0.9078	−1.346	0.699	−1.925	0.0652
Pink	1	5.48	0.0272	0.641	0.274	2.340	0.0272
Residuals	26						

**Table 2:** Final reduced model (LM) describing the effects of snake morphology and color on beauty ranking.

	**Df**	***F*-value**	**Pr(>*F*)**	**Estimate**	**SE**	***t*-value**	**Pr(>|*t*|)**
Intercept				2.529	0.340	7.449	< 0.0001
Pattern	1	78.53	< 0.0001	−7.422	2.334	−3.180	0.0035
Mean lightness	1	3.04	0.0917	−2.307	0.671	−3.436	0.0018
SD Lightness	1	2.40	0.1320	−2.082	0.661	−3.152	0.0038
White	1	5.41	0.0273	0.998	0.254	3.932	0.0005
Black	1	9.02	0.0055	−0.617	0.220	−2.799	0.0090
Red	1	22.46	< 0.0001	0.345	0.073	4.739	< 0.0001
Residuals	29						

### Explanation of perceived fear by the snake's real dangerousness

The presented snake species also differ in their dangerousness for humans. We compared the specific level of threat to humans (the dangerousness category, see the Materials and methods and Supplementary Table 2) with the species' fear evaluation. In both the Czech and Azerbaijani respondents, the perceived fear significantly correlated with the real threat (Spearman correlation coefficient; Czechs: *r* = 0.716, *p* < 0.0001; Azerbaijanis: *r* = 0.725, *p* < 0.0001), but the perceived beauty did not (Spearman correlation, Czechs: *r* = 0.227, *p* = 0.1767; Azerbaijanis: *r* = 0.160, *p* = 0.3430).

## Discussion

### Comparison of fear and beauty evaluations of snakes between czech and azerbaijanis

In this paper, our main aim was to make cross-cultural comparisons of human fear responses to various venomous and non-venomous snake species common in Europe, Middle East, and North Africa. We hypothesized differences in fear perception of snakes in the Czech Republic compared to Azerbaijan, the two countries with considerably different risks of envenoming. While snakes in the Czech Republic do not present a significant threat, the risk of serious injuries or even deaths caused by snakebites in Azerbaijan is much higher. Contrary to our expectations, in the sample consisted largely of college students, we found a significant cross-cultural agreement in fear evaluation (*r*^2^ = 0.65, *p* < 0.0001). This is in agreement with our previous study demonstrating that despite differences in snake abundance and risk of envenoming people from different countries self-report comparable snake fear (Polák et al., 2016).

To some extent, the Czechs and Azerbaijanis were able to categorize the snake species according to the real threat to humans. Most of the envenomings in Azerbaijan are caused by one particular species, the Levant viper, an aggressive snake that is responsible for many deaths. We predicted that it should be seen by Azerbaijanis as more fearful than other snakes. Despite that, the Levant viper did not figure at the top of fear ranking (14th most fearful out of 36 species). By contrast, only one slightly venomous snake that lives in the Czech Republic, the common European adder, was seen as quite dangerous by the Czech respondents (4th most fearful out of 36 species). Other deadly venomous species that are similar to the Levant viper (see Supplementary Figure 4), and thus present a serious threat, are evaluated by both the Azerbaijani and Czech respondents as highly fear eliciting. Compared to vipers, slender bodied elapids, including the Egyptian cobra in a resting position, were perceived as less fear eliciting. This could mean the fear module responds to a broader and more general category of dangerous, fear eliciting snakes rather than particular species. This is especially important from the ethnobiological and conservational perspective as due to the presence of one dangerous snake species people may incline to killing all other snakes that are similar though less dangerous. From the psychological point of view, it is interesting to find out how broad this “snake” category is (see below and in section Attitude Toward Snakes).

Moreover, when we analyzed the differences in fear evaluation between men and women from both countries in more details, the species causing slight discrepancies are not the most dangerous snakes of Azerbaijan, but other viperids (controls) that live neither in Azerbaijan nor the Czech Republic. Thus, co-habitation with the highly dangerous Levant viper in Azerbaijan did not lead to increased fear elicited by this species. The respondents rather generalized the fear evaluation of the local dangerous model snake to all vipers and pit vipers resembling it. Another possibility is that the characteristic viper-like body shape, i.e., a triangular head, narrow neck, and a thick body, is a prototypical stimulus that humans recognize ancestrally. This is because in Eastern Africa, the place of human origins, our ancestors have probably lived for last two million years together with highly venomous viperid species (Isbell, 2006). Nowadays, this region is inhabited by many deadly venomous species that still cause human deaths like the North-East carpet viper (*Echis pyramidum*), African puff adder (*B. arietans*), rhinoceros viper (*Bitis nasicornis*), rhombic night adder (*Causus rhombeatus*), or the African hairy bush viper (*Atheris hispida*) (Spawls et al., 1995). To tackle this question, research must be conducted on other ethnics where meeting a snake is still risky, but viperids do not pose the major threat.

The colubroid snakes evolved a highly efficient venom delivery system probably in the Late Cretaceous (around 60 Myr; Vidal and Hedges, 2002), with viperids evolving in Asia in the Early Miocene (around 20 Myr) or even later (Wüster et al., 2008). Molecular data show the diversification of some genera within viperids, e.g., *Vipera* and *Macrovipera*, around 10 Myr (Pook et al., 2009). Primates, including human ancestors, have also evolved predominantly in the Old World (switching between Africa and SE Asia), i.e., they continuously occurred in sympatry with the viperids and other venomous snakes. Subsequently, the mankind evolved in Africa from its common ancestor with chimpanzees around 7–13 million years ago (Langergraber et al., 2012). Thus, the last 10 million years our direct ancestors have spent in Africa, initially in tropical forests and later in open savannas (Dawkins, 2005). The repeated expansion of modern humans out of the African continent to Eurasia, where they could encounter the current genera of viperids, is estimated to about 90–100 Kyr to Levant, 60 Kyr to western Asia, and 40 Kyr to Europe (Deshpande et al., 2009; Stewart and Stringer, 2012). Thus, contemporary human populations have been exposed to various local snake faunas representing a different risk of envenoming. It can be expected that the number of deaths/injuries caused annually by snakebites in some countries is not a sufficient selective pressure to create an evolutionary adaptation to recognize and fear specific snake species in a given region (here the Levant viper in Azerbaijan) within such an evolutionary short time scale. A hypothetical innate adaptation of humans to the local snake species would need to evolve during a couple thousands of years following the African migration, which is unlikely. On the other hand, the co-evolution with viperids have been long enough to form a general mental representation of dangerous viperid snakes associated with a high fear response.

We have also tested human perception of snake beauty that, as we expected based on our previous findings and contrary to fear, should demonstrate intercultural similarity. We have already shown that in Papua New Guinea which is inhabited by highly venomous snakes from the Elapidae family (O'Shea, 1996), villagers evaluate the beauty of boid snakes similarly to Czech students (Marešová et al., 2009a); the cross-cultural agreement on beauty ranking was relatively high (*r*^2^ = 0.76). This is despite an entirely different cross-cultural background of the Papuans who are exposed to a higher envenoming risk than people in Central Europe. Moreover, the viperids are missing in Papua New Guinea, so the morphotype of local dangerous snakes is different. A broader cross-cultural agreement (*r*^2^ ranging from 0.56 up to 0.92) on beauty of these snakes was also found between the Papuans and villagers from Bolivia, Philippines, India (Rajasthan and Dehli), Malawi, and Morocco (Frynta et al., 2011). In this paper, we provide support for such findings showing a comparable or even higher cross-cultural agreement in the snake beauty evaluation (*r*^2^ = 0.83, *p* < 0.0001).

### Relationship between perceived fear and beauty of snakes

We suppose that asthetic evaluation of snake beauty is closely connected with joy (Kühn and Gallinat, 2012; Frynta et al., 2014) and that during ranking according to fear this emotion is triggered as well. Previously, we have found only a loose correlation between perceived beauty and fear in king snakes when respondents evaluated photographs but not the same live specimens (Landová et al., 2012). King snakes include brightly colored species mimicking deadly venomous coral snakes and the perception of beauty and fear were rather two independent processes in these snakes. Interestingly, in this study, where respondents evaluate a more diverse sample containing snakes from 29 genera and 6 families, we also found that snake species eliciting higher fear tend to be at the same time perceived as more beautiful by all nationality/sex sub groups except Azerbaijani women. However, this correlation between fear and beauty evaluations is lower and varies cross-culturally (Czech men: *r*^2^ = 0.42; Czech women: *r*^2^ = 0.30; and Azerbaijan men: *r*^2^ = 0.17), so people are able to subjectively distinct these two dimensions during ranking of the snake photographs. If we found, on the other hand, a high agreement between the fear and beauty rankings, it would mean that there is a need for simple explicit categorization rules that help respondents deal with both tasks promptly and are not dependent on a particular evaluation.

### Features predicting evaluation of snake species as fearful or beautiful

The most fear evoking trait on snakes was a specific body form, a short snake with a wide but distinct neck and big eyes. On the contrary, worm-like snakes from the Typhlopidae family did not elicit fear in neither of the countries (see Figure 2). It has been shown that the typical curvilinear shape enhances quick detection of the snake (LoBue, 2014) even if it is introduced in scenes in gray scale (Hayakawa et al., 2011). Azerbaijani, as well as Czech respondents, perceive as the most fear evoking stimulus the Egyptian cobra in a threatening posture (with the extended neck and in an upright position, see Supplementary Figure 4D). However, the same species in a resting position (control) was according to fear only 22nd out of 36 species in Azerbaijani and 28th out of 36 species in Czech respondents. Thus, the snake's position seems crucial for its fear evaluation. Only this particular species was included in two different positions as the threatening cobras are very well known from the media, while threatening positions of other snakes are not so apparent. However, the snake's position before an attack (S-shaped neck) communicates a willingness to behave defensively (Johnson, 1975) and is worth of interest if people that are unfamiliar with snake behavior are able to recognize it as a signal of danger and perceive consequently more fear.

The high congruence in beauty perception may be partly explained not only by asthetic preferences for colors (Crozier, 1999), symmetry (Enquist and Arak, 1994), and contrast patterns on animals (Marešová et al., 2009b; Lišková et al., 2015), but also by more delicate features like animal body shapes (Lišková and Frynta, 2013; Janovcová, 2015). We have previously found in king snakes that perception of beauty depends on the body weight and presence of red and black colors (Landová et al., 2012). King snakes include a great variety of color forms, some possessing aposematic patterns (red-black-yellow/white stripes) that are highly preferred by respondents. However, in other studies, this color pattern contributes to perceiving the animal as dangerous (Prokop and Fančovičová, 2013). The set of snakes used in this paper does not comprise brightly colored species, thus the pattern complexity, high lightness, and presence of black color (inherently presented in contrast patterns) increase the relative beauty of presented species. Thus, the contrasting pattern on snake body is considered as beautiful similarly to our results in boid snakes (Marešová and Frynta, 2008) or interestingly in pitta birds (Lišková et al., 2015).

### Attitude toward snakes

According to the contemporary theory in social psychology, learning can account for most of the attitudes we hold. We know that attitudes can be implicitly acquired via classical, operant, or evaluative conditioning. Formation of implicit attitudes employs the processes of both classical and operant conditioning, but social learning is probably of the same importance (Wegener Carlston and Carlston, 2005). Moreover, people can also form attitudes explicitly on the spot with just a little cognitive effort using several heuristics (Schwarz, 2000). It is generally accepted that attitudes form future behavior and results from a meta-analysis by Glasman and Albarracín (2006) indicate that this effect is stronger when attitudes are easy to recall and stable over time.

In our study, the respondents also filled out a questionnaire about their attitude toward snakes. Although most of our subjects were students and employees of biological sciences colleges, this relationship was different in both countries. In the Czech Republic, the proportion of people who like snakes (54%) is similar to those who hate them (46%). However, the majority of the Azerbaijani respondents have either a neutral relationship toward snakes (49%) or hate them (37%). This is an interesting finding regarding the association between biology education and more positive attitudes toward animals (see also section Study Limitations). Despite more biology students being represented in the Azerbaijani sample, their attitudes were more negative than those of Czechs. Thus, their education did not overcome the aversion to snakes. The number of unpleasant encounters should be theoretically an explanation for the different relationship to snakes, as only five species of snakes live in the Czech Republic, whilst about 32 snake species occur in Azerbaijan. Nevertheless, our results show that encounters with snakes are comparably common in both countries (87 and 99%). Different attitudes toward snakes are related to another question; if the respondent has ever killed a snake or has seen someone killing it.

It is known that dangerous animals, especially snakes, are killed for safety reasons in different geographical regions, e.g., in Brazil (Alves et al., 2009, 2012), India (Balakrishnan, 2010), or Nepal (Pandey et al., 2016). In Azerbaijan, a half of the respondents (49%) have seen killing a snake or killed one themselves (Supplementary Figure 5 shows a dead Levant viper killed by villagers in Katex, northern Azerbaijan, found during a zoological field expedition). By contrast, only a few Czechs have ever killed a snake (14%), either as a part of research or involuntarily (driving over a snake with a car). This proportion of reported cases of snake killing by adults is consistent with previously published data on children who intended to kill a snake if they would encounter one (Ballouard et al., 2013). This was surprisingly high among children from Morocco (45%), Portugal (60%), or even Slovakia (90%). However, in other countries this willingness to kill a snake was lower, ranging from 29% in Turkey to 7–13% in six European countries. Moreover, this was influenced by the sex of the respondents and their fear or likability of snakes (Ballouard et al., 2013). As Makashvili et al. (2014) reported, negative attitude to snakes can be overcome with knowledge.

### Does attitude toward snakes explains beauty or fear evaluation?

The attitude toward snakes explains perceived fear of snakes only marginally in the Czech respondents. Only 6% of women and 3% of men have purely negative attitudes to snakes, e.g., dislike and fear them. However, any cross-cultural differences in fear evaluation of snakes are mainly due to the different fear evaluation of snakes in Azerbaijan women. Higher fear response and more negative attitudes toward snakes in women have been reported several times (Arrindell et al., 2003; Prokop et al., 2009). Although we confirmed that Azerbaijani women in our sample had more negative attitudes toward snakes (26% highly dislike snakes) this does not explain their differences in relative fear evaluation. Moreover, ranking according to beauty was fully independent regardless of personal attitudes toward snakes.

### Study limitations

More than a half of our respondents from both countries were university biology students. One might argue that concerning their type of education and young age they did not constitute a proper representative sample. For example, Schlegel and Rupf (2010) found that natural resource sciences students showed higher levels of affinity to animals. Bjerke and Østdahl (2004) reported a positive correlation between the respondent's educational level in general and positive attitudes toward animals. In this study, this might be true for the attitude toward snakes, however, our results, and previous studies showed that the effect of individual characteristics on the ranking of animal species according to their beauty or perceived fear was usually only marginal (Frynta et al., 2010), even when comparing such different populations as Czech students and villagers in Papua New Guinea (Marešová et al., 2009a). Moreover, Collins (1976) pointed out that obvious differences between the first-year students (represented the most in our study) and biology majors could be found. The students we have recruited were mainly from the first or second year of university. At this stage of curriculum they have still not done any specialized course in herpetology. We also hypothesize that in the case of salient, emotionally charged, and evolutionary relevant stimuli, such as snakes, the stimulus' characteristics may be more important for its evaluation than sociodemographic traits of the respondent. This is consistent with results of Prokop and Tunnicliffe (2008), who found a moderate correlation between attitude toward and knowledge of bats, but not spiders. They suggested that this may be partly influenced by greater fear of spiders compared to fear of bats. To conclude, although we are aware of the limitations and want to avoid over-generalization of our results, we believe that our sample was adequate for the purpose of this study.

Throughout the study, we have found discrepancies between the Azerbaijani women and the rest of the subject groups (Azerbaijani men and Czech men and women) on almost all measures. Based on an evolutionary hypothesis, women have a higher reproductive value compared to men, because they give birth to the progeny. In the evolutionary history of mankind, women were staying around their homes gathering food while men went out hunting. Doing so, women had more chances of encountering snakes lurking in the environment. Having to protect not only themselves, but also their children or even unborn babies, there was a good evolutionary reason for women to develop higher fear of snakes compared to men (Öhman et al., 2012). Even today, women suffer considerably more often from specific animal phobias than men (Fredrikson et al., 1996). In light of the above, the results obtained from the Azerbaijani women make sense. Nevertheless, one has to explain then why no similar gender difference has been found in the Czech Republic. Perhaps, it may be argued that the traditional gender roles associated with differences in the expression of fear, among others, are more pronounced in Azerbaijan, where 98% of the population are Muslims. Contrary to that, the mostly atheist Czech population has gone through sociodemographic changes in the last few decades resulting in equalization of gender roles and stereotypes.

## Summary and conclusion

The major portion of variability in the data explains high similarity in fear 65% (as well as beauty 83%) perception of snakes in the Czech Republic compared to Azerbaijan, the two countries with considerably different risks of envenoming. The rest of the variability reflects the differences that should be accounted to some respondents' individual characteristics (especially the Azerbaijani women being different in fear evaluation of the snake species). Interestingly, biology education of the respondents influenced only the attitude to toward snakes, but not the fear evaluation itself. These results are further demonstrated by the canonical DFA showing the overlap between nationality/sex groups. The differences are caused by different evaluation of only three snake species specific for fear and beauty ranking.

Snake beauty evaluation was a valuable control task, to be sure that respondents rank the pictures according to elicited fear, not only due to applying more general esthetic and categorization rules. We found only a slight correlation between fear and beauty, indicating that only some species were evaluated as both fearful and beautiful at the same time. Further, we detected a different set of attributes behind the evaluation of fear (mainly the snake's morphology) and beauty (mainly colors and pattern) of snakes with only the lightness affecting both. In both countries, the respondents evaluated the Egyptian cobra in a threatening position or the “viper” morphotype as the most fear eliciting and dangerous. However, they did not evaluate the cobra in a resting position nor the particular species that locally represent the major risk of snakebite (the common European adder in Czech Republic and the Levant viper in Azerbaijan) as the most fear eliciting. The generalization concept of viperids as the most fearful snakes in our study may be due to their co-evolutionary history with humans and is responsible for a high cross-cultural agreement. We also found the role of experience (killing a snake or seeing someone killing a snake) on fear evaluation, but not on beauty evaluation of snakes. A general attitude (likability) of snakes was influenced by the family membership, but the influence of attitude or family factor on relative fear or beauty evaluations was not significant. Thus, it seems that the family context, environment where one grows up, may change the way we view snakes, whether we like them or not, but it will not affect the perceived fear or beauty of particular snake species which might be a universally shared pattern.

Despite the differences in the attitude toward snakes between the Azerbaijani and Czech respondents, agreement in the evaluation of snake beauty and fear is cross-culturally high. The “viper” type is generalized as the most fear evoking appearance of a snake in both countries. Thus, the fear response to snakes is not directly explainable by the observed current environmental and cultural differences.

## Author contributions

EL, DF, and AG: conceived and designed the research; NB, MJ, EL, SP, MS, and JP: performed the research; DF and MJ: analyzed the data; EL, DF, MJ, and JP: wrote the paper; EL, DF, MJ, JP, NB, AG, MS, and SP: revised the paper and approved the final version.

### Conflict of interest statement

The authors declare that the research was conducted in the absence of any commercial or financial relationships that could be construed as a potential conflict of interest.
