# Supplementary material for: Association Between Fear and Beauty Evaluation of Snakes: Cross-Cultural Findings
Source: Front Psychol. 2018 Mar 16;9:333. doi: 10.3389/fpsyg.2018.00333 (PMC5865084; doi:10.3389/fpsyg.2018.00333)
Supplement: Supplementary file 1 [file Table1.docx]

***Supplementary Material***

**Association Between Fear and Beauty Evaluation of Snakes: Cross-cultural Findings**

Eva Landová^*^, Natavan Bakhshaliyeva, Markéta Janovcová, Šárka Peléšková, Mesma Suleymanova, Jakub Polák, Akif Guliev, Daniel Frynta^*^

*** Correspondence:** Eva Landová: [evalandova@seznam.cz](mailto:evalandova@seznam.cz), Daniel Frynta: [frynta@centrum.cz](mailto:frynta@centrum.cz)

**Supplementary Table 1. Information about snake species included in the testing set.**

| **Species** | **Common name** | **Family** | **Distribution** | **Length** | **Source** |
| --- | --- | --- | --- | --- | --- |
| *Atractaspis engaddensis* | Israeli mole viper | Lamprophiidae | no | 685 | El Din, 2006 |
| *Bitis arietans* | African puff viper | Viperidae | no | 1 400 | Marais, 2004 |
| *Cerastes cerastes* | Desert horned viper | Viperidae | no | 800 | Egan, 2007 |
| *Coluber rhodorachis* | Jan's cliff racer | Colubridae | no | 1 280 | Egan, 2007 |
| *Coronella austriaca* | Smooth snake | Colubridae | Cz, Az | 920 | Stojanov et al., 2011 |
| *Dolichophis jugularis* | Large whip snake | Colubridae | no | 1 415 | Wallach et al., 2014 |
| *Echis coloratus* | Palestine saw-scaled viper | Viperidae | no | 750 | Egan, 2007 |
| *Eirenis collaris* | Collared dwarf racer | Colubridae | Az | 316 | Bannikov et al., 1977 |
| *Elaphe quatuorlineata* | Four-lined ratsnake | Colubridae | no | 1 400 | Schultz, 1996 |
| *Eryx jaculus* | Javelin sand boa | Boidae | Az | 530 | Wallach et al., 2014 |
| *Eryx jayakari* | Arabian sand boa | Boidae | no | 640 | Egan, 2007 |
| *Gloydius halys* | Halys pit viper | Viperidae | Az | 714 | Wallach et al., 2014 |
| *Hemorrhois nummifer* | Asian racer | Colubridae | no | 2 000 | Valakos et al., 2008 |
| *Hemorrhois ravergieri* | Spotted whip snake | Colubridae | Az | 1 180 | Bannikov et al., 1977 |
| *Macroprotodon cucullatus* | False smooth snake | Colubridae | no | 650 | Coborn, 1991 |
| *Macrovipera lebetina* | Levant viper | Viperidae | Az | 1 600 | Bannikov et al., 1977 |
| *Malpolon monspessulanus* | Montpellier snake | Lamprophiidae | Az | 2 500 | Valakos et al., 2008 |
| *Micrelaps muelleri* | Müller's black-headed snake | Lamprophiidae | no | 370 | Amr et al., 1997 |
| *Montivipera xanthina* | Coastal viper | Viperidae | no | 1 500 | Stojanov et al., 2011 |
| *Naja haje* | Egyptian cobra | Elapidae | no | 2 500 | Spawls et al., 2002 |
| *Natrix natrix* | European grass snake | Colubridae | Cz, Az | 2 050 | Stojanov et al., 2011 |
| *Natrix tessellata* | Dice snake | Colubridae | Cz, Az | 1 300 | Bannikov et al., 1977 |
| *Platyceps najadum* | Dahl's whip snake | Colubridae | Az | 1 500 | Stojanov et al., 2011 |
| *Platyceps ventromaculatus* | Glossy-bellied racer | Colubridae | no | 950 | Egan, 2007 |
| *Pseudocerastes persicus* | Persian horned viper | Viperidae | Az | 688 | Khan, 2002 |
| *Rhagerhis moilensis* | Moila snake | Lamprophiidae | no | 1 890 | Egan, 2007 |
| *Rhynchocalamus melanocephalus* | Palestine kukri snake | Colubridae | Az | 480 | Egan, 2007 |
| *Spalerosophis diadema* | Diadem snake | Colubridae | no | 1 550 | El Din, 2006 |
| *Telescopus dhara* | Arabian cat snake | Colubridae | no | 1 300 | Spawls et al., 2002 |
| *Telescopus fallax* | European cat snake | Colubridae | Az | 1 000 | Coborn, 1991 |
| *Vipera ammodytes* | Nose-horned viper | Viperidae | Az | 1 100 | Stojanov et al., 2011 |
| *Vipera berus* | Commnon European viper | Viperidae | Cz | 1 040 | Stojanov et al., 2011 |
| *Vipera ursinii* | Meadow viper | Viperidae | no | 600 | Valakos et al., 2008 |
| *Walterinnesia aegyptia* | Black desert cobra | Elapidae | no | 1 400 | Egan, 2007 |
| *Xerotyphlops vermicularis* | European blind snake | Typhlopidae | Az | 450 | Bannikov et al., 1977 |
| *Zamenis situla* | European ratsnake | Colubridae | no | 1 000 | Schultz, 1996 |

The first three columns state the Latin and English common name and the taxonomic family of the species. “Distribution”: Az = species occurs in Azerbaijan, Cz = species occurs in the Czech Republic, no = species does not live in either of the studied countries; „Length“ is the maximum body length in millimeters; „Source“ is the source of information on body length.

There are 32 species of snakes in the Caspian region in Azerbaijan (cf. Alekperov, 1977), nine of them are venomous and represent a serious risk. The most dangerous snake for human (in term of bite risk, envenoming and even death) is the highly venomous Levant viper *Macrovipera lebetina*, a larger viper of up to 160 cm in length (Bannikov et al., 1977) delivering 150 mg of venom in one bite (Valenta, 2008). The other venomous species (the Caucasian pit viper *Gloydius halys caucasicus*, Radde's viper *Montivipera raddei*, Wagner's viper *Montivipera wagneri*, Persian horned viper *Pseudocerastes persicus*, nosed-horned viper *V. ammodytes*, Dinnik's viper *Vipera dinniki*, Shemakhan viper *Vipera shemakhensis*, and the Transcaucasian long-nosed viper *Vipera transcaucasiana*) cause injuries only accidentally (Bakhshaliyeva, unpublished data). They are found in Azerbaijan in smaller densities (Frynta, personal communication) or inhabit smaller areas than the Levant viper *M. lebetina* (Alekperov, 1977).
