# Supplementary material for: Association Between Fear and Beauty Evaluation of Snakes: Cross-Cultural Findings
Source: Front Psychol. 2018 Mar 16;9:333. doi: 10.3389/fpsyg.2018.00333 (PMC5865084; doi:10.3389/fpsyg.2018.00333)
Supplement: Supplementary file 2 [file Table2.docx]

***Supplementary Material***

**Association Between Fear and Beauty Evaluation of Snakes: Cross-cultural Findings**

Eva Landová^*^, Natavan Bakhshaliyeva, Markéta Janovcová, Šárka Peléšková, Mesma Suleymanova, Jakub Polák, Akif Guliev, Daniel Frynta^*^

*** Correspondence:** Eva Landová: [evalandova@seznam.cz](mailto:evalandova@seznam.cz), Daniel Frynta: [frynta@centrum.cz](mailto:frynta@centrum.cz)

**Supplementary Table 2. The dangerousness of the tested species for humans.**

| **Species** | **Venom** | **Dangerous** | **Category** | **LD50 IV** | **Source** |
| --- | --- | --- | --- | --- | --- |
| *Atractaspis engaddensis* | yes | yes | 4 | 0.6-0.75 | Weiser et al., 1984 |
| *Bitis arietans* | yes | yes | 5 | 0.4-2.0 | Brown, 1973 |
| *Cerastes cerastes* | yes | yes | 4 | 0.95 | Abdel–Aal et al., 2014 |
| *Coluber rhodorachis* | no | no | 1 | - | - |
| *Coronella austriaca* | no | no | 1 | - | - |
| *Dolichophis jugularis* | yes | no | 2 | NA | NA |
| *Echis coloratus* | yes | yes | 5 | 2.88 | Samy et al., 2015 |
| *Eirenis collaris* | no | no | 1 | - | - |
| *Elaphe quatuorlineata* | yes | no | 2 | NA | NA |
| *Eryx jaculus* | no | no | 1 | - | - |
| *Eryx jayakari* | no | no | 1 | - | - |
| *Gloydius halys* | yes | yes | 4 | 9.75 | Spawls and Branch, 1995 |
| *Hemorrhois nummifer* | no | no | 1 | - | - |
| *Hemorrhois ravergieri* | yes | no | 1 | NA | NA |
| *Macroprotodon cucullatus* | yes | no | 1 | NA | NA |
| *Macrovipera lebetina* | yes | yes | 5 | 7.58 | Nalbantsoy et al., 2012 |
| *Malpolon monspessulanus* | yes | yes | 3 | 6.5 | Weinstein et al., 2011 |
| *Micrelaps muelleri* | yes | no | 2 | NA | NA |
| *Montivipera xanthina* | yes | yes | 4 | 8.78 | Nalbantsoy, 2013 |
| *Naja haje* | yes | yes | 5 | 0.495 | Abdel–Aal et al., 2014 |
| *Natrix natrix* | yes | no | 2 | NA | NA |
| *Natrix tessellata* | yes | no | 2 | 25.0 | Weinstein et al., 2011 |
| *Platyceps najadum* | no | no | 1 | - | - |
| *Platyceps ventromaculatus* | no | no | 1 | - | - |
| *Pseudocerastes persicus* | yes | yes | 5 | 16.2 | Yousefkhani et al., 2014 |
| *Rhagerhis moilensis* | yes | no | 2 | NA | NA |
| *Rhynchocalamus melanocephalus* | no | no | 1 | - | - |
| *Spalerosophis diadema* | yes | no | 2 | 2.75 | Hossie et al., 2013 |
| *Telescopus dhara* | yes | no | 2 | NA | NA |
| *Telescopus fallax* | yes | no | 2 | NA | NA |
| *Vipera ammodytes* | yes | yes | 4 | 1.2 | Brown, 1973 |
| *Vipera berus* | yes | yes | 4 | 0.55 | Mallow et al., 2003 |
| *Vipera ursinii* | yes | yes | 4 | 1.0 | Steinhoff, 2017 |
| *Walterinnesia aegyptia* | yes | yes | 5 | 0.48 | Spawls and Branch, 1995 |
| *Xerotyphlops vermicularis* | no | no | 1 | - | - |
| *Zamenis situla* | yes | no | 2 | NA | NA |

The first two columns show whether the snake has a venom delivery system and is potentially dangerous for humans; „Category“ is the level of threat for human life: 5 = venomous and very dangerous, 4 = venomous and considerably dangerous, 3 = venomous and potentially dangerous, 2 = venomous but not dangerous, 1 = non-venomous snake; „LD50 IV“ is the 50% lethal dose of venom intravenously in mg/kg mice, „Source“ is the source of LD50 IV values.
