# Supplementary material for: Association Between Fear and Beauty Evaluation of Snakes: Cross-Cultural Findings
Source: Front Psychol. 2018 Mar 16;9:333. doi: 10.3389/fpsyg.2018.00333 (PMC5865084; doi:10.3389/fpsyg.2018.00333)
Supplement: Supplementary file 3 [file Table3.DOCX]

***Supplementary Material***

**Association Between Fear and Beauty Evaluation of Snakes: Cross-cultural Findings**

Eva Landová^*^, Natavan Bakhshaliyeva, Markéta Janovcová, Šárka Peléšková, Mesma Suleymanova, Jakub Polák, Akif Guliev, Daniel Frynta^*^

*** Correspondence:** Eva Landová: [evalandova@seznam.cz](mailto:evalandova@seznam.cz), Daniel Frynta: [frynta@centrum.cz](mailto:frynta@centrum.cz)

**Supplementary Table 3.** An English translation of the questionnaire used in the study, however, the respondents completed it in their native language (Czech or Azeri).

| **Surname** | |  | |
| --- | --- | --- | --- |
| **Sex male/female** | |  | |
| **Age** | |  | |
| **Relationship to snakes (on scale 1 to 7:**  1 - I like them, I would like to breed them and keep them at home; 4 - neutral; 7 - I don't like them, I hate/fear them | |  | |
| **I keep snakes at home.** **Yes / No** (If the answer is  Yes, write what species you keep) | |  | |
| **Have you encountered snakes in the real life?**  If the answer is Yes, how and where have you met snakes? | |  | |
| **Have you ever killed a snake? Have you seen**  **somebody killing a snake?** Yes/No | |  | |
| **Your curriculum,** your education (primary,  secondary, high school) and field of study  (biological, technical, medical, agricultural, social  sciences), **what is your profession?** | |  | |
| **E-mail** | |  | |
| **Signature:** I confirm hereby that I participated on this study voluntarily and agree for the data to be used exclusively for scientific purposes. The anonymity of data is guaranteed. I also agree that I may be contacted in the future by email to complete some information about myself or to take part in the following projects. | |  | |
| **Beauty** (from the most to the least beautiful) | | **Fear** (from the most to the least feared) | |
| 1 | 20 | 1 | 20 |
| 2 | 21 | 2 | 21 |
| 3 | 22 | 3 | 22 |
| 4 | 23 | 4 | 23 |
| 5 | 24 | 5 | 24 |
| 6 | 25 | 6 | 25 |
| 7 | 26 | 7 | 26 |
| 8 | 27 | 8 | 27 |
| 9 | 28 | 9 | 28 |
| 10 | 29 | 10 | 29 |
| 11 | 30 | 11 | 30 |
| 12 | 31 | 12 | 31 |
| 13 | 32 | 13 | 32 |
| 14 | 33 | 14 | 33 |
| 15 | 34 | 15 | 34 |
| 16 | 35 | 16 | 35 |
| 17 | 36 | 17 | 36 |
| 18 | 37 | 18 | 37 |
| 19 | X | 19 | X |
