# Supplementary material for: Association Between Fear and Beauty Evaluation of Snakes: Cross-Cultural Findings
Source: Front Psychol. 2018 Mar 16;9:333. doi: 10.3389/fpsyg.2018.00333 (PMC5865084; doi:10.3389/fpsyg.2018.00333)
Supplement: Supplementary file 4 [file Table4.DOCX]

***Supplementary Material***

**Association Between Fear and Beauty Evaluation of Snakes: Cross-cultural Findings**

Eva Landová^*^, Natavan Bakhshaliyeva, Markéta Janovcová, Šárka Peléšková, Mesma Suleymanova, Jakub Polák, Akif Guliev, Daniel Frynta^*^

*** Correspondence:** Eva Landová: [evalandova@seznam.cz](mailto:evalandova@seznam.cz), Daniel Frynta: [frynta@centrum.cz](mailto:frynta@centrum.cz)

**Supplementary Table 4.** List of authors of the photographs used in the testing set together with the photos sources.

| **Species** | **Family** | **Photograph author** | **Source** |
| --- | --- | --- | --- |
| *Atractaspis engaddensis* | Lamprophiidae | Moshe Klukowski | <https://www.flickr.com/photos/54302606@N07/14003549544> |
| *Bitis arientans* | Viperidae | David Bygott | <http://www.tablemountain.net/blog/entry/table-mountain-for-nature-lovers> |
| *Cerastes cerastes* | Viperidae | Baudilio R. Fernándes | [https://www.flickr.com/photos/budilord/5700080197/77Fys](https://www.flickr.com/photos/budilord/5700080197/in/photolist-9FGpfM-bSnLt8-c3PgTJ-4tNGxS-bqyRS7-dBy1fi-bqyRyj-6jz8UJ-agwY2M-bqyTAs-6juUEr-di2odx-demrqT-78y2F3-dQMTyY-di2oFk-qSHkBS-62hxWt-demsVg-9nK1xy-demrEw-k3aXCX-o4hqKu-fhtpxd-di2np3-di2ooD-ppVn9v-eqkPmW-di2o8p-di2oyt-9nGhpT-p8qbz2-8u9rbD-agmXP3-v7n4oX-vLBwNq-w1V29o-demL8L-k2eiDB-bDtLXB-bqyPLo-4ZgC9K-6cjTwD-bqySDb-b5YZ6X-gnFt6d-cs9ccs-bqyTah-bqyQmS-77Fys) |
| *Coluber (Platyceps) rhodorachis* | Colubridae | Moshe Klukowski | <https://www.flickr.com/photos/54302606@N07/8404824555> |
| *Coronella austriaca* | Colubridae | B. Kahl | Coborn (1991) |
| *Dolichophis jugularis* | Colubridae | Eyal Bartov | <http://www.eyalbartov.com/ISRAEL/Reptiles-Amphibian-זוחלים-ודוח/ReptilesofIsrael/Snakes/i-dccCtnZ/A> |
| *Echis coloratus* | Viperidae | Moshe Klukowski | <https://www.flickr.com/photos/54302606@N07/6815281903> |
| *Eirenis collaris* | Colubridae | Omid Mozaffari | <https://commons.wikimedia.org/wiki/File:Eirenis_collaris_by_Omid_Mozaffari_3.jpg> |
| *Elaphe quatuorlineata* | Colubridae | G. Kreiner | Valacos et al. (2008) |
| *Eryx jaculus* | Boidae | Moshe Klukowski | <https://www.flickr.com/photos/54302606@N07/5029262834> |
| *Eryx jayakari* | Boidae | Damien Egan | Egan (2007) |
| *Gloydius halys* | Viperidae | Petr Szymonik | <http://www.fotoszymon.wbs.cz/Gloydius-hallys-carraganus-p-stepni.html?1&souborfotky=bartek1-101pbl.jpg#foto> |
| *Hemorrhois nummifer* | Colubridae | O. Attum | Baha El Din (2006) |
| *Hemorrhois ravergieri* | Colubridae | Omid Mozaffari | <http://www.reptarium.cz/taxonomy/Hemorrhois-ravergieri/36512> |
| *Macroprotodon cucullatus* | Colubridae | Moshe Klukowski | <https://www.flickr.com/photos/54302606@N07/7115471127> |
| *Macrovipera lebetina* | Viperidae | Omid Mozaffari | [https://www.flickr.com/photos/omid_mozaffari/14116878921/](https://www.flickr.com/photos/omid_mozaffari/14116878921/in/photolist-nxwrSk-neg6gf-nvsGha-8aThGf-8aQ1AK-8aQ1z2-ne7DEv-KNtzyo-LKzwKg) |
| *Malpolon monspessulanus* | Lamprophiidae | El Hammam | Baha El Din (2006) |
| *Micrelaps muelleri* | Lamprophiidae | Moshe Klukowski | <https://www.flickr.com/photos/54302606@N07/5028553923> |
| *Montivipera xanthina* | Viperidae | Benny Trapp | <https://en.wikipedia.org/wiki/Vipera_xanthina#/media/File:Benny_Trapp_Montivipera_xanthina.jpg> |
| *Naja haje* (threat) | Elapidae | Damien Egan | Egan (2007) |
| *Naja haje* (resting) | Elapidae | Damien Egan | Egan (2007) |
| *Natrix natrix* | Colubridae | Todd Pierson | <http://www.discoverlife.org/mp/20p?see=I_TPN3901&res=640> |
| *Natrix tessellata* | Colubridae | Damien Egan | Egan (2007) |
| *Platyceps najadum* | Colubridae | Dr. S. Minton | Coborn (1991) |
| *Platyceps ventromaculatus* | Colubridae | Damien Egan | Egan (2007) |
| *Pseudocerastes persicus* | Viperidae | Jonathan Gropp | <https://www.flickr.com/photos/gropp/4216434892> |
| *Rhagerhis moilensis* | Lamprophiidae | Damien Egan | Egan (2007) |
| *Rhynchocalamus melanocephalus* | Colubridae | Damien Egan | Egan (2007 |
| *Spalerosophis diadema* | Colubridae | Dr. S. Minton | Coborn (1991) |
| *Telescopus dhara* | Colubridae | J. Wisser | Coborn (1991) |
| *Telescopus fallax* | Colubridae | Moshe Klukowski | [https://www.flickr.com/photos/54302606@N07/6155439757/](https://www.flickr.com/photos/54302606@N07/6155439757/in/photolist-anWeMF-fZuvf6-5RCR1c-puF6Q7-gs2xHh-4dX74R-4e2cbs-qV4asr-5RCQQi-78rUVH-76tCcc-fomqnJ-fnM8aL-dixu35-78rUXB-8pPQRd-d3cXyG-c2kaVJ-ciwW5o-9Fg4oB-835yNH-dgvTG2-d3cW7d-dixtY1-4e2cK9-qHS9Gi-etuqdp-4dXdYp-nbiLz5-dgvQMa-d3cWP9-rdVyAg-nbd9nm-4dXdjp-cqaXRW-fHuMuy-4e2rZ5-4dXekr-4e2sGY-d3cRAS-5qsLNV-6Fadcw-6mzjz-835yUi-Jb934h-bof7KM-6F64GH-6mzjA-cix8du-anwwbf) |
| *Vipera ammodytes* | Viperidae | R. T. Zappalorti | Coborn (1991) |
| *Vipera berus* | Viperidae | Mark Robinson | <https://www.flickr.com/photos/66176388@N00/3893228734/> |
| *Vipera ursinii* | Viperidae | Aurelio Candido | [https://www.flickr.com/photos/terzocchio/7248000190](https://www.flickr.com/photos/terzocchio/7248000190/in/photolist-c3tTWw-c3tUj5-cxZebs-c3tVg1-cxZfrd-dCgqcB-hSxtEx-vSDwrD-cKWJU1-c3tUNL-yF4pHU-dgkyRT-yF4n2m-zWqU3u-dgkyKg-dpiL4p-dpiVbq-cKWB8Q-dM4M8k-zbFEdU-gquP3G-eyqjWt-fHh4UE-czUQMb-fGZuuB-qU3jBT-gquPhQ-9qCqAn-duhRH2-9A1Mbo-dHbLFp-fHh46f-oEsJeD-fGZuf8-qRTmUU-9zXPaT-eJTWZe-aDkYZs-a6QzMr-pXbtk1-dCvnuq-a6TrMA-a6Trz1-a6TrFA-jznE8o-onXWW1-ge5zmh-a6QzD2-dmrFXC-a6QzAM)/ |
| *Walterinnesia aegyptia* | Elapidae | Ltshears | <https://en.wikipedia.org/wiki/File:Sinai-Desert-Cobra.jpg> |
| *Xerotyphlops vermicularis* | Typhlopidae | R. Sindaco | Baha El Din (2006) |
| *Zamenis situla* | Colubridae | E. Razzetti | Valacos et al. (2008) |
