# Supplementary material for: Association Between Fear and Beauty Evaluation of Snakes: Cross-Cultural Findings
Source: Front Psychol. 2018 Mar 16;9:333. doi: 10.3389/fpsyg.2018.00333 (PMC5865084; doi:10.3389/fpsyg.2018.00333)
Supplement: Supplementary file 5 [file Table5.DOCX]

***Supplementary Material***

**Association Between Fear and Beauty Evaluation of Snakes: Cross-cultural Findings**

Eva Landová^*^, Natavan Bakhshaliyeva, Markéta Janovcová, Šárka Peléšková, Mesma Suleymanova, Jakub Polák, Akif Guliev, Daniel Frynta^*^

*** Correspondence:** Eva Landová: [evalandova@seznam.cz](mailto:evalandova@seznam.cz), Daniel Frynta: [frynta@centrum.cz](mailto:frynta@centrum.cz)

**Supplementary Table 5. Results of fear and beauty evaluations.**

|  | | | **Fear** | | | **Beauty** | |
| --- | --- | --- | --- | --- | --- | --- | --- |
| **Species** | **Cz** | | **Az** | **Cz** | | **Az** | |
| *Atractaspis engaddensis* | 0.7681 | | 0.6732 | 0.9742 | | 0.9412 | |
| *Bitis arietans* | 0.5987 | | 0.6454 | 0.6409 | | 0.5318 | |
| *Cerastes cerastes* | 0.4178 | | 0.5473 | 0.6692 | | 0.0856 | |
| *Coluber rhodorachis* | 0.9717 | | 0.9847 | 0.7509 | | 0.8384 | |
| *Coronella austriaca* | 0.8431 | | 0.8022 | 0.7987 | | 0.7571 | |
| *Dolichophis jugularis* | 0.9113 | | 0.7927 | 0.8934 | | 0.9589 | |
| *Echis coloratus* | 0.5462 | | 0.5731 | 0.7411 | | 0.7700 | |
| *Eirenis collaris* | 1.0696 | | 1.0304 | 0.9877 | | 0.8837 | |
| *Elaphe quatuorlineata* | 0.9278 | | 0.7081 | 0.7598 | | 0.6848 | |
| *Eryx jaculus* | 0.7934 | | 0.8683 | 0.7887 | | 0.7112 | |
| *Eryx jayakari* | 0.9601 | | 0.8213 | 1.1058 | | 1.0598 | |
| *Gloydius halys* | 0.6520 | | 0.7087 | 0.7034 | | 0.7331 | |
| *Hemorrhois nummifer* | 0.7097 | | 0.8648 | 0.7941 | | 0.8594 | |
| *Hemorrhois ravergieri* | 0.8709 | | 0.8773 | 0.7151 | | 0.6826 | |
| *Macroprotodon cucullatus* | 0.8225 | | 0.7086 | 0.7932 | | 0.6327 | |
| *Macrovipera lebetina* | 0.6518 | | 0.7293 | 0.8407 | | 0.7992 | |
| *Malpolon monspessulanus* | 0.8270 | | 0.6929 | 0.9853 | | 1.0167 | |
| *Micrelaps muelleri* | 0.8078 | | 0.9107 | 0.7443 | | 0.7242 | |
| *Montivipera xanthina* | 0.5171 | | 0.7108 | 0.5085 | | 0.5806 | |
| *Naja haje (threat-posture)* | 0.2350 | | 0.3054 | 0.7235 | | 0.7747 | |
| *Naja haje* | 0.9288 | | 0.8541 | 0.8451 | | 0.9367 | |
| *Natrix natrix* | 0.9713 | | 0.8581 | 0.6474 | | 0.5664 | |
| *Natrix tessellata* | 0.8442 | | 0.7421 | 0.9466 | | 0.8760 | |
| *Platyceps najadum* | 1.0511 | | 0.9637 | 0.7438 | | 0.9384 | |
| *Platyceps ventromaculatus* | 0.9331 | | 0.9592 | 0.7330 | | 0.8079 | |
| *Pseudocerastes persicus* | 0.4122 | | 0.4709 | 0.8726 | | 1.0251 | |
| *Rhagerhis moilensis* | 0.9604 | | 0.8759 | 0.8009 | | 0.7618 | |
| *Rhynchocalamus melanocephalus* | 1.0038 | | 1.0044 | 0.8126 | | 0.8274 | |
| *Spalerosophis diadema* | 0.8213 | | 0.9033 | 0.7167 | | 0.7477 | |
| *Telescopus dhara* | 0.8350 | | 0.8812 | 0.9376 | | 0.9059 | |
| *Telescopus fallax* | 0.7514 | | 0.7358 | 0.6107 | | 0.6335 | |
| *Vipera ammodytes* | 0.4912 | | 0.7336 | 0.4748 | | 0.4857 | |
| *Vipera berus* | 0.5091 | | 0.7233 | 0.4944 | | 0.3153 | |
| *Vipera ursinii* | 0.6597 | | 0.8697 | 0.6165 | | 0.5857 | |
| *Walterinnesia aegyptia* | 0.7143 | | 0.5833 | 0.8550 | | 0.8383 | |
| *Xerotyphlops vermicularis* | 1.4128 | | 1.1083 | 1.4868 | | 1.4677 | |
| *Zamenis situla* | 0.8583 | | 0.8388 | 0.5466 | | 0.5944 | |

Evaluation of fear and beauty as the mean of square-root arcsin-transformed data. „Cz“ = Czech respondents, „Az“ = Azerbaijani respondents.
