# Supplementary material for: Association Between Fear and Beauty Evaluation of Snakes: Cross-Cultural Findings
Source: Front Psychol. 2018 Mar 16;9:333. doi: 10.3389/fpsyg.2018.00333 (PMC5865084; doi:10.3389/fpsyg.2018.00333)
Supplement: Supplementary file 6 [file Table6.DOCX]

***Supplementary Material***

**Association Between Fear and Beauty Evaluation of Snakes: Cross-cultural Findings**

Eva Landová^*^, Natavan Bakhshaliyeva, Markéta Janovcová, Šárka Peléšková, Mesma Suleymanova, Jakub Polák, Akif Guliev, Daniel Frynta^*^

*** Correspondence:** Eva Landová: [evalandova@seznam.cz](mailto:evalandova@seznam.cz), Daniel Frynta: [frynta@centrum.cz](mailto:frynta@centrum.cz)

**Supplementary Table 6.** Factor loadings for the canonical DFA of fear evaluation.

| **Species** | **Root 1** | **Root 2** | **Root 3** |
| --- | --- | --- | --- |
| *Vipera ursinii* | 0.488350 | -0.083467 | -0.071940 |
| *Montivipera xanthina* | 0.370311 | 0.128756 | -0.407022 |
| *Elaphe quatuorlineata* | -0.396897 | -0.080300 | 0.112898 |
| *Echis coloratus* | 0.005604 | 0.430607 | -0.057414 |
| *Natrix tessellata* | -0.184469 | -0.369048 | 0.315762 |
| *Vipera berus* | 0.352907 | 0.076021 | 0.380830 |
| *Hemorrhois nummifer* | 0.300471 | 0.048720 | 0.296850 |
| *Coluber rhodorachis* | -0.043718 | 0.345064 | 0.185224 |
| *Micrelaps muelleri* | 0.131015 | 0.068202 | 0.300154 |
| *Gloydius halys* | 0.149861 | -0.143292 | -0.133247 |
| *Eirenis collaris* | -0.033588 | -0.209768 | -0.066290 |
| *Xerotyphlops vermicularis* | -0.350005 | -0.088013 | 0.149350 |
| *Walterinnesia aegyptia* | -0.160278 | -0.123787 | -0.158916 |
| *Natrix natrix* | -0.170593 | 0.033077 | -0.166317 |
| *Rhageris moilensis* | -0.143880 | -0.226577 | 0.180531 |
| *Dolichophis jugularis* | -0.217340 | 0.070648 | 0.131822 |
| *Malpolon monspesulanus* | -0.265240 | -0.157173 | -0.068192 |
