# Supplementary material for: Association Between Fear and Beauty Evaluation of Snakes: Cross-Cultural Findings
Source: Front Psychol. 2018 Mar 16;9:333. doi: 10.3389/fpsyg.2018.00333 (PMC5865084; doi:10.3389/fpsyg.2018.00333)
Supplement: Supplementary file 7 [file Table7.DOCX]

***Supplementary Material***

**Association Between Fear and Beauty Evaluation of Snakes: Cross-cultural Findings**

Eva Landová^*^, Natavan Bakhshaliyeva, Markéta Janovcová, Šárka Peléšková, Mesma Suleymanova, Jakub Polák, Akif Guliev, Daniel Frynta^*^

*** Correspondence:** Eva Landová: [evalandova@seznam.cz](mailto:evalandova@seznam.cz), Daniel Frynta: [frynta@centrum.cz](mailto:frynta@centrum.cz)

**Supplementary Table 7.** Factor loadings for the canonical DFA of beauty evaluation.

| **Species** | **Root 1** | **Root 2** | **Root 3** |
| --- | --- | --- | --- |
| *Vipera berus* | 1.9828 | -1.00652 | -0.85888 |
| *Macroprotodon cuccullatus* | 1.5769 | 1.72321 | -0.53188 |
| *Telescopus dhara* | 1.9897 | -0.03178 | -0.34703 |
| *Elaphe quatuorlineata* | 1.6132 | 0.25938 | 0.50578 |
| *Hemorrhois ravergieri* | 1.2863 | 0.70821 | -0.67524 |
| *Eirenis collaris* | 1.6092 | -1.04807 | 0.59368 |
| *Platyceps najadum* | -0.9306 | -0.77942 | -0.82307 |
| *Hemorrhois nummifer* | -0.6099 | -2.30913 | 0.08246 |
| *Natrix tessellata* | 0.8861 | 1.45391 | 0.61815 |
| *Gloydius halys* | -0.5264 | -0.86398 | -1.19439 |
| *Bitis arietans* | 0.7583 | -0.35458 | 1.78807 |
| *Cerastes cerastes* | 0.5326 | 0.33332 | 0.85442 |
| *Micrelaps muelleri* | 0.4726 | 0.50956 | 1.08679 |
| *Vipera ursinii* | 1.2445 | 0.16034 | 0.63233 |
| *Vipera ammodytes* | 0.6694 | -1.17961 | -1.21429 |
| *Rhageris moilensis* | 1.0183 | -1.27475 | -0.33907 |
| *Walterinnesia aegyptia* | 1.1478 | -0.99022 | 0.92289 |
| *Eryx jayakari* | 0.6165 | -1.23696 | 0.93158 |
| *Macrovipera lebetina* | 0.8628 | -1.62269 | 0.61360 |
| *Natrix natrix* | 0.913 | -0.98195 | -0.36238 |
| *Eryx jaculus* | 0.2456 | -0.91001 | -0.98304 |
| *Coronella austriaca* | 0.7577 | -0.94374 | 0.27319 |
| *Rhynchocalamus melanocephalus* | 0.2588 | -0.81950 | -0.02455 |
| *Xerotyphlops vermicularis* | 0.1992 | 1.21118 | -0.45125 |
