# Supplementary figures and images for: Association Between Fear and Beauty Evaluation of Snakes: Cross-Cultural Findings
Source: Front Psychol. 2018 Mar 16;9:333. doi: 10.3389/fpsyg.2018.00333 (PMC5865084; doi:10.3389/fpsyg.2018.00333)

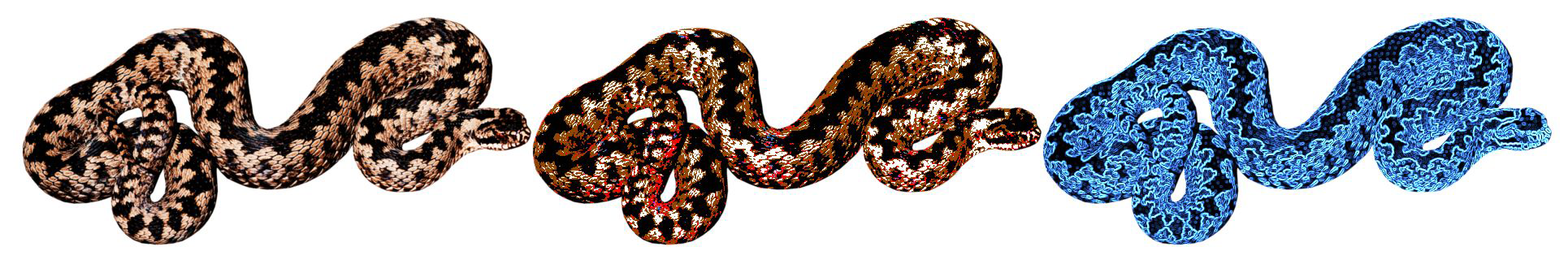

Supplement: Supplementary file 8 [file Image1.TIF]

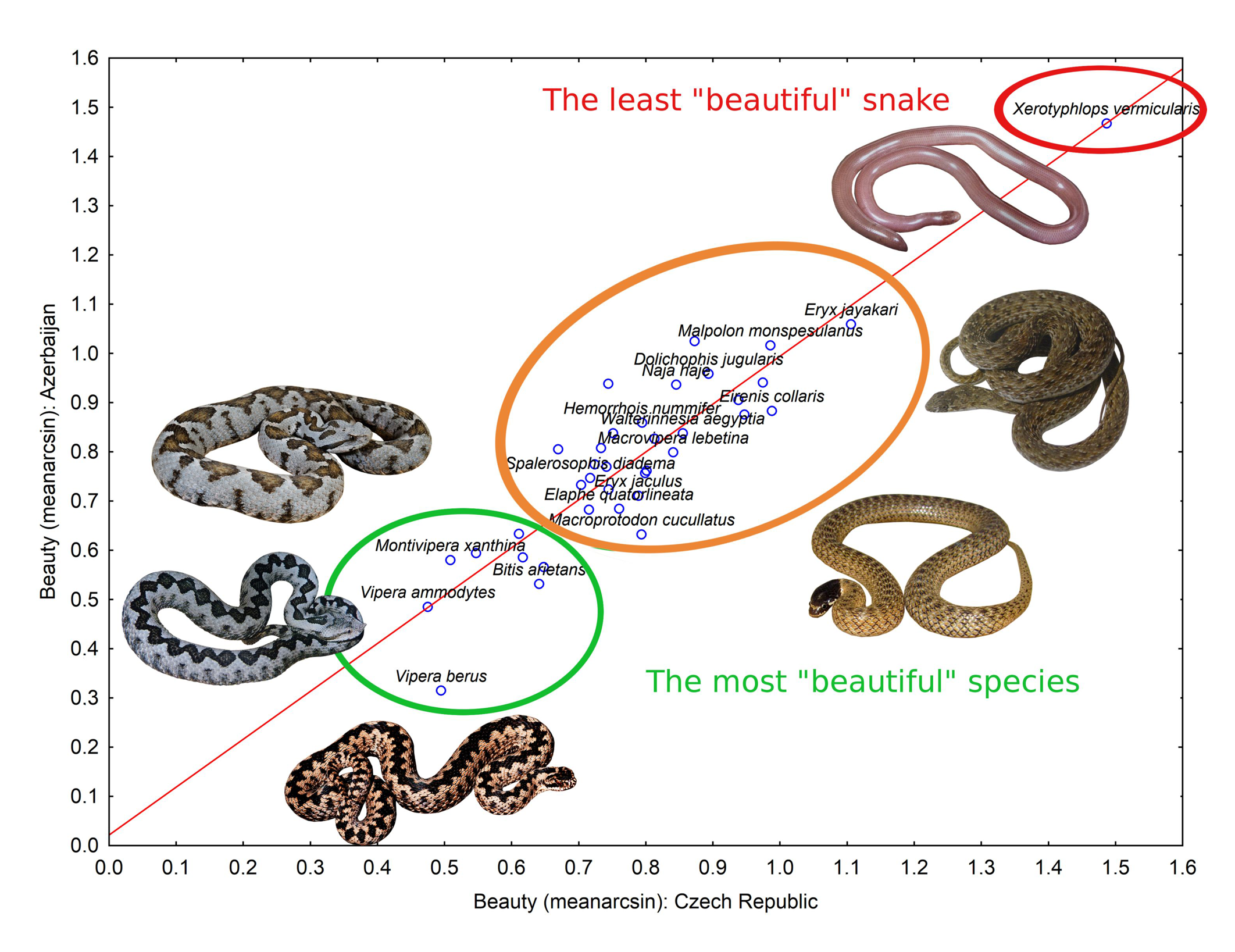

Supplement: Supplementary file 9 [file Image2.TIF]

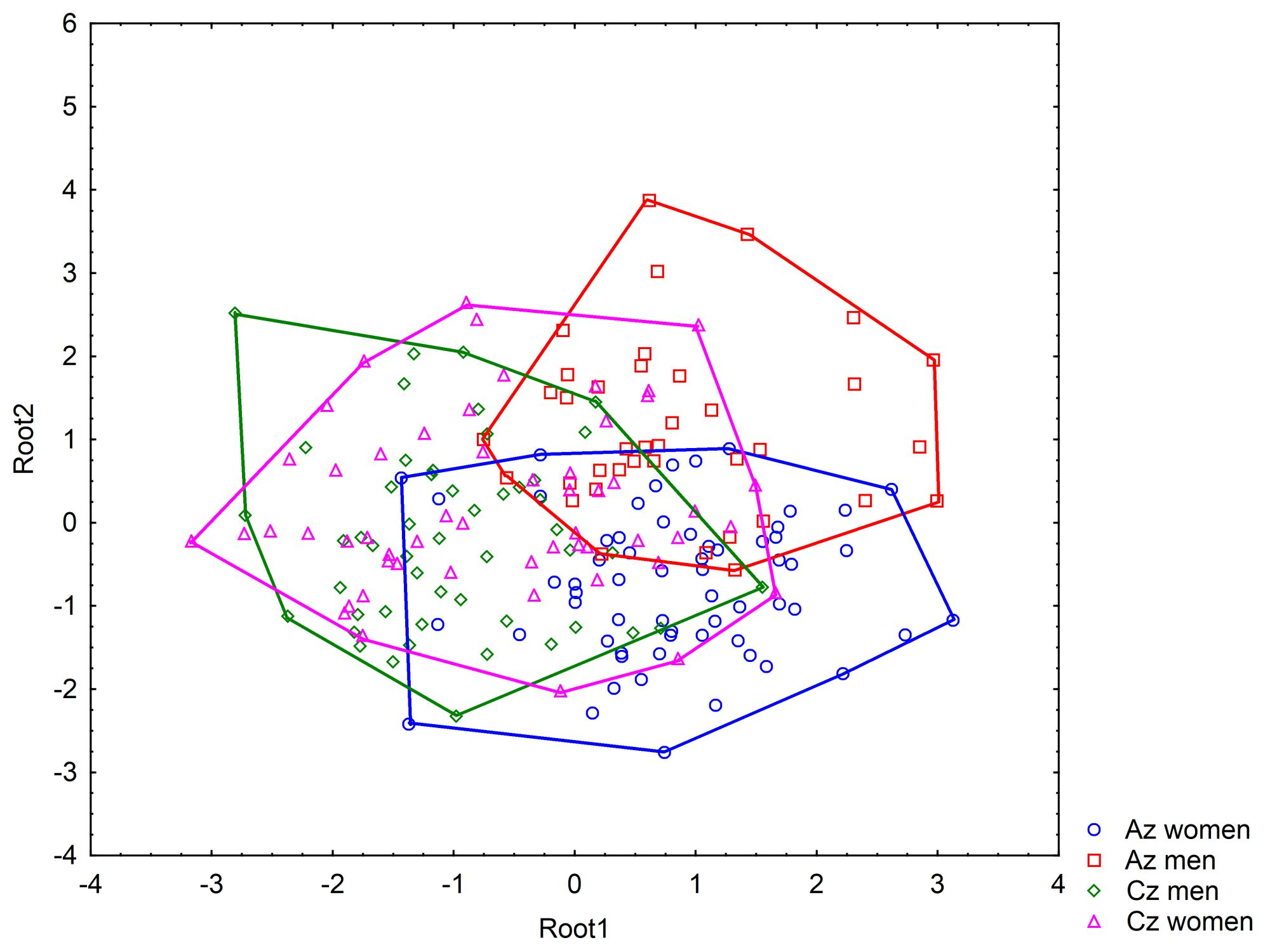

Supplement: Supplementary file 10 [file Image3.TIF]

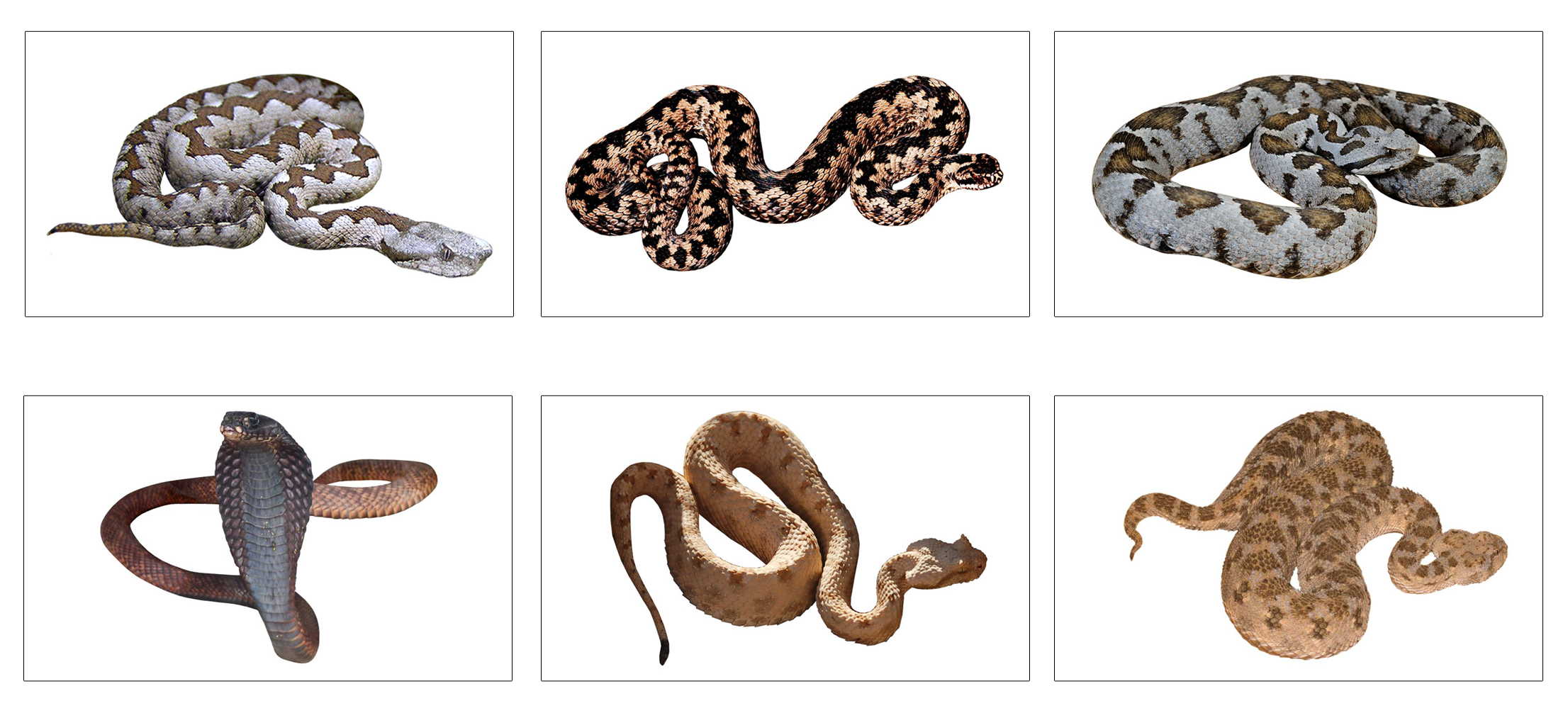

Supplement: Supplementary file 11 [file Image4.TIF]

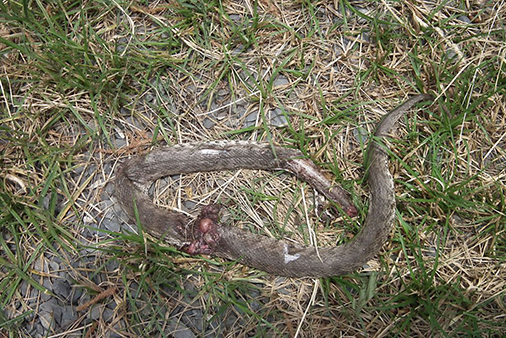

Supplement: Supplementary file 12 [file Image5.TIF]
