# Supplementary material for: Association Between Fear and Beauty Evaluation of Snakes: Cross-Cultural Findings
Source: Front Psychol. 2018 Mar 16;9:333. doi: 10.3389/fpsyg.2018.00333 (PMC5865084; doi:10.3389/fpsyg.2018.00333)
Supplement: Supplementary file 13 [file DataSheet1.docx]

***Supplementary Material***

**Association Between Fear and Beauty Evaluation of Snakes: Cross-cultural Findings**

Eva Landová^*^, Natavan Bakhshaliyeva, Markéta Janovcová, Šárka Peléšková, Mesma Suleymanova, Jakub Polák, Akif Guliev, Daniel Frynta^*^

*** Correspondence:** Eva Landová: [evalandova@seznam.cz](mailto:evalandova@seznam.cz), Daniel Frynta: [frynta@centrum.cz](mailto:frynta@centrum.cz)

**Supplementary Figure 1.** A graphical output from the Barvocuc program. It contains the original analyzed photo and then an image with detected colors according to our setting and pattern complexity. The pattern complexity is calculated as the boundary between areas of two different colors. Species in the picture: the common European viper *V. berus*, original photo: Mark Robinson.

Supplementary Figure 2. A correlation of beauty evaluations from the Czech Republic and Azerbaijan. The graph was calculated from square-root arcsin-transformed data, i.e., the lower the value the more beautiful the species is according to the respondents. Pearson correlation coefficient *r*^2^ = 0.816, p < 0.0001.

**Supplementary Figure 3.** The canonical DFA of beauty evaluation. Similarly to fear evaluation, this analysis also showed significant differences between the sexes and countries when comparing the Azerbaijani women to all other groups and the Azerbaijani men to the Czech respondents. However, the plot of the first two canonical factors showed a considerable overlap of the groups for beauty evaluation and a low classification success (63%). A forward stepwise procedure selected only 24 species from 36, out of which those corresponding the best with the discrimination criteria (i.e., the largest Wilks’ lambda) were the false smooth snake (*Macroprotodon cucullatus*; Wilks’ lambda = 0.4428, p < 0.0001), the common European adder (*Vipera berus*; Wilks‘ lambda = 0.4313, p = 0.0002), and the collared dwarf racer (*Eirenis collaris*; Wilks’ lambda = 0.4248, p = 0.0009). It means that the sex and cross-cultural differences in beauty evaluation are mainly due to a different evaluation of three species out of the 36 tested.

**Supplementary Figure 4.** Three the most beautiful and fearful species. In the upper line, the first two species (**A**, **B**) were perceived as the most beautiful in both countries, the one on the right (**C**) was evaluated as the third most beautiful in the Czech Republic. In the lower line, the species perceived as the most fearful in both countries are presented (**D**, **E**, **F**). Photo: (**A**) the nosed-horned viper *V. ammodytes*, original photo Mircea Nita; (**B**) the common European viper *V. berus*, original photo Mark Robinson, (**C**) the coastal viper *M. xanthina*, original photo Benny Trapp; (**D**) the Egyptian cobra *N. haje*, original photo Milan Kaftan, (**E**) the Persian horned viper *P. persicus*, original photo Jonathan Gropp; (**F**) the desert horned viper *C. cerastes*, original photo Mino Zig.

**Supplementary Figure 5.** Killing snakes in Azerbaijan. A dead Levant viper (*M. lebetina*) killed by villagers that was found during a field expedition in Katex, northern Azerbaijan, photo: Barbora Kaftanová.
